# Supplementary material for: Encoding of speech modes and loudness in ventral precentral gyrus
Source: Nat Commun. 2026 Apr 15;17:5301. doi: 10.1038/s41467-026-71284-4 (PMC13270037; doi:10.1038/s41467-026-71284-4)
Supplement: Supplementary file 1 — Supplementary Information [file 41467_2026_71284_MOESM1_ESM.pdf]

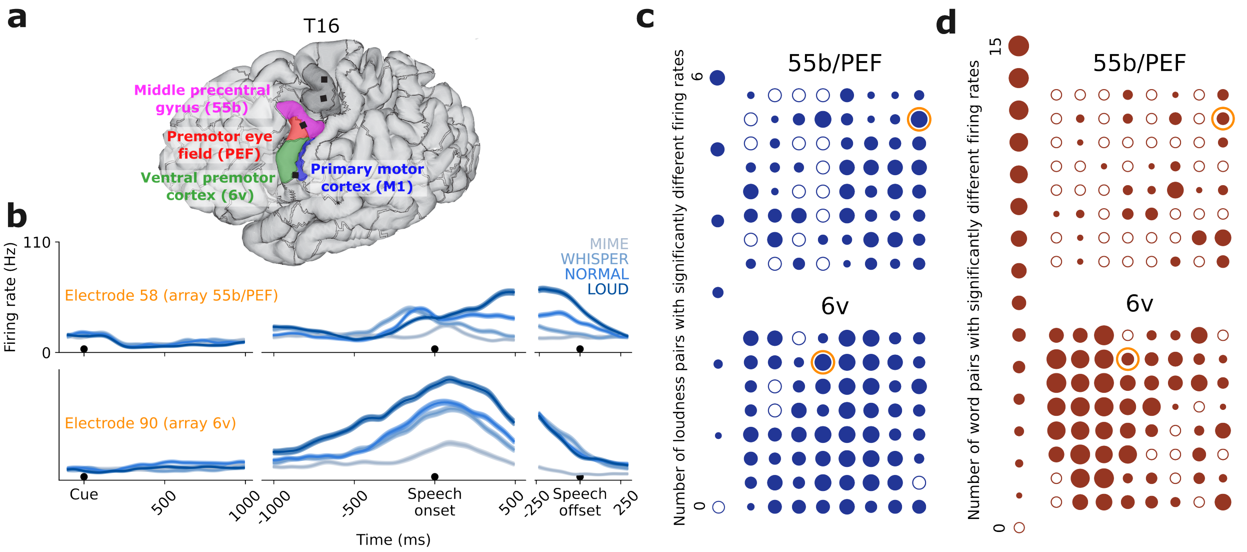

Supplementary Figure 1. Loudness and word encoding across participant T16's vPCG. a. 3D reconstruction of T16's brain, showing the locations of the Utah arrays (black squares) and relevant brain regions estimated from fMRI. The two most dorsal arrays in the hand motor cortex were not analyzed in this study. **b.** Firing rates (mean ± s.e.) from an example electrode in each array, computed by trial-averaging within loudness conditions. Activity is aligned to both cue onset (left), speech onset (middle) and speech offset (right). All arrays exhibited loudness-related modulation, i.e. had some electrodes tuned to loudness levels. **c.** Electrodes tuned to attempted speech loudness level, determined by significant differences in firing rates between loudness conditions (one-way ANOVA with post-hoc Tukey's honestly significant difference test, $p < 0.05$). **d.** Electrodes tuned to attempted words determined by significant differences in firing rates between words (one-way ANOVA with post-hoc Tukey's honestly significant difference test, $p < 0.05$). Electrodes whose firing rates are shown in (b) are marked with orange circles.

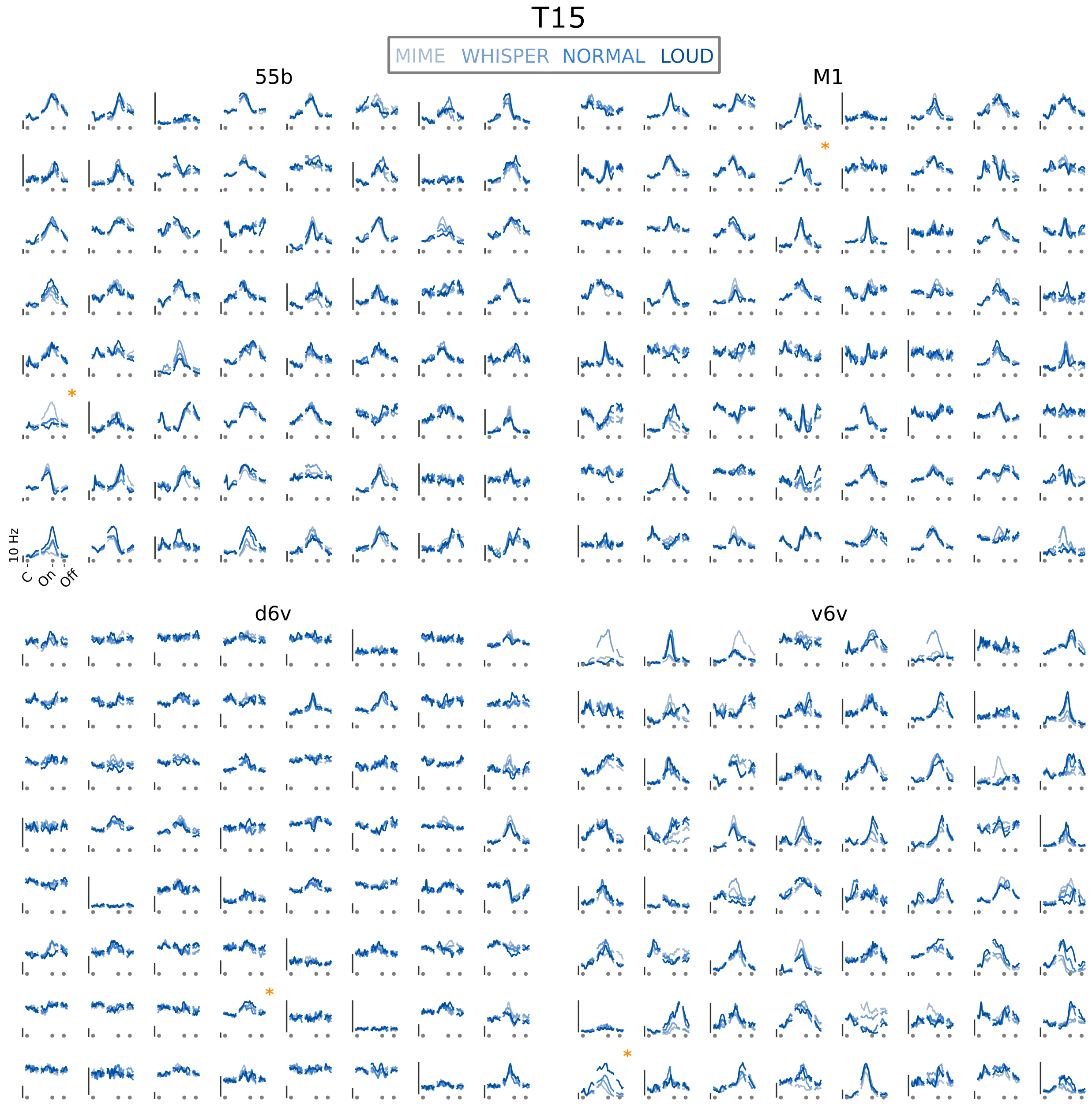

Supplementary Figure 2. All electrodes' firing rates for T15. Firing rates per electrode were computed by trial-averaging within each loudness condition. Neural activity was aligned to cue onset ('C', left), speech onset ('On', middle), and speech offset ('Off', right). Electrodes exhibit different patterns of modulation across vPCG. The four electrodes shown as examples in Fig. 1 are marked with gold stars.

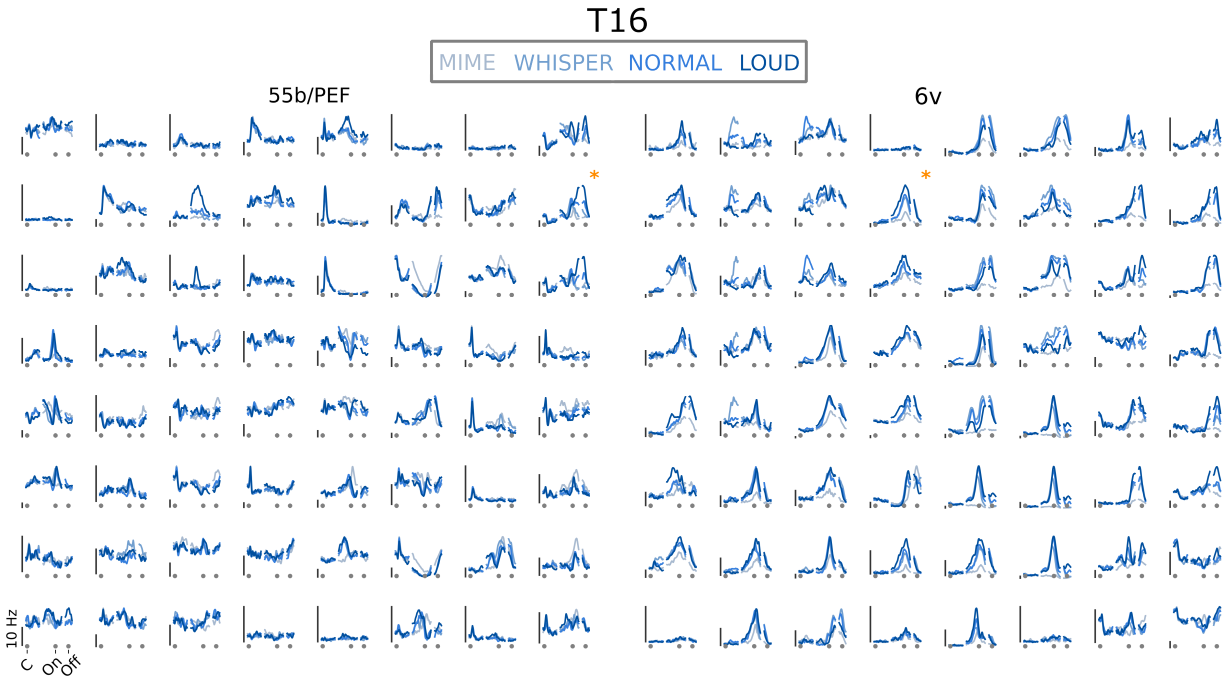

Supplementary Figure 3. All electrodes' firing rates for T16. Firing rates per electrode were computed by trial-averaging within each loudness condition. Neural activity was aligned to cue onset ('C', left), speech onset ('On', middle), and speech offset ('Off', right). Electrodes exhibit different patterns of modulation across vPCG. The two electrodes shown as examples in Supp. Fig. 1 are marked with gold stars.

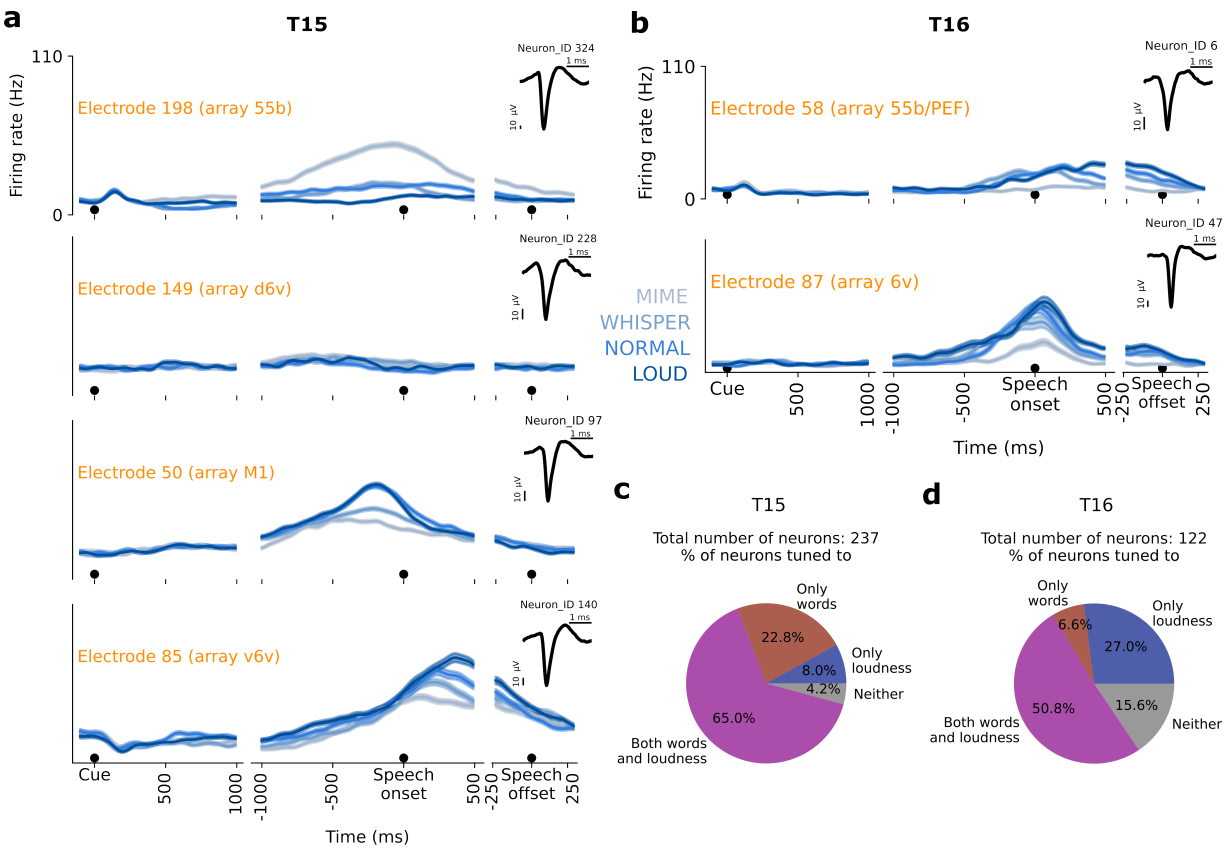

Supplementary Figure 4: Single-unit activity and task tuning. a,b) Firing rates (mean ± s.e.) from an example neuron recorded from each array for participants T15 and T16, respectively. Insets show mean action potential waveforms of these neurons. **c,d)** Proportion of neurons tuned to different task parameters (word, loudness, both, or neither) for T15 and T16, respectively. Tuning was determined by measuring, for each neuron, significant differences in firing rates across loudness conditions and across word conditions (one-way ANOVA with post-hoc Tukey's honestly significant difference test, $p < 0.05$).

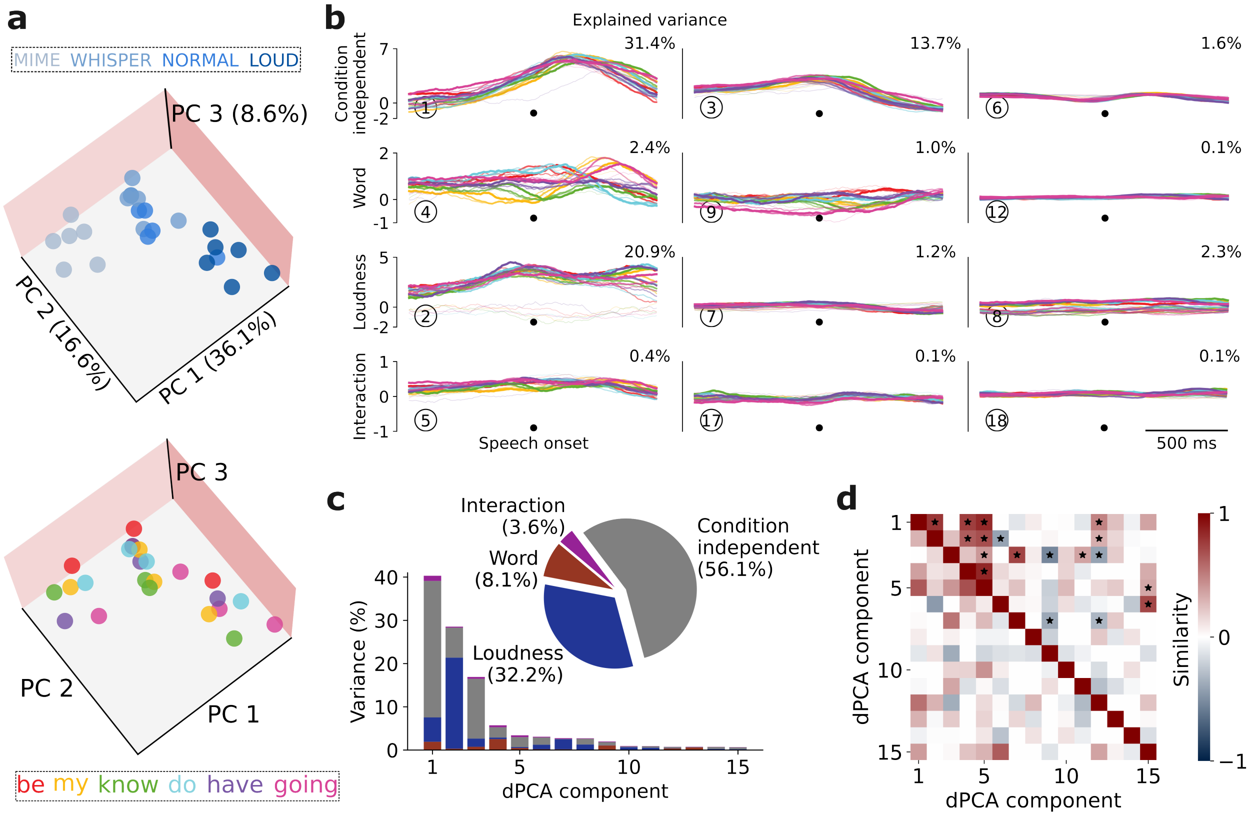

Supplementary Figure 5. T16's neural ensemble activity separably encodes loudness from words. a. PCA projections of T16's trial-averaged spike band power from [-750, 750] ms around speech onset during the word-loudness task. Both subplots show the same data projections but with conditions colored according to loudness (top) or which word was spoken (bottom) to illustrate the independent encoding of loudness versus phonemic content. **b.** dPCA applied to these same data. Each subplot shows the data projected onto the respective dPCA decoder axis. Each plot contains 24 curves (4 loudness levels × 6 words), with loudness represented by increasing saturation and linewidth from MIME up to LOUD, and words shown in different colors. **c.** Explained variance of individual demixed PCs. The pie chart illustrates the proportion of total neural variance attributed to each task parameter. **d.** Relationship between demixed PCs. The upper right triangle shows the dot product between all pairs of the first 15 demixed principal axes, and the lower left triangle shows the correlations between these components. Stars indicate pairs that are significantly and robustly non-orthogonal.

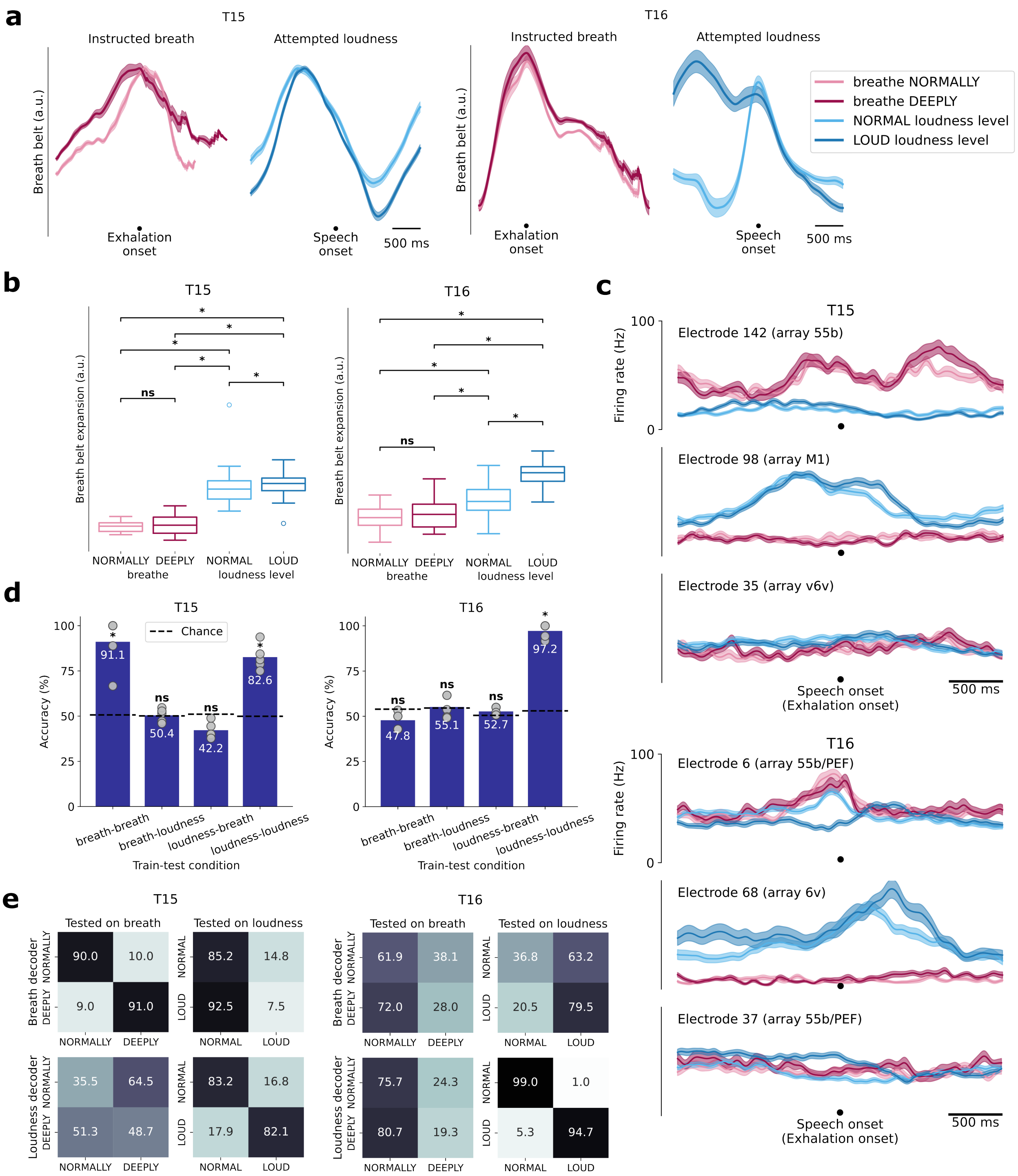

Supplementary Figure 6: Breath is modulated with loudness level, but a neural breath decoder does not predict attempted loudness. a. Breath belt expansion and compression (mean ± s.e.) during the instructed breathing task (left) and sentence-loudness task (right) for each participant. Breath depth differed between attempted loudness and instructed breathing, but there was little change between breathing normally and deeply, which is consistent with the participants reporting that they found it difficult to perform this task. **b.** Breath expansion statistics for instructed breathing and speech tasks. Box plots depict the median (center line), 25th and 75th

percentiles (lower and upper boundaries of the box), 25th and 75th percentiles ± 1.5 times the interquartile range (whiskers), and outliers (circles). Breath belt expansion significantly differed between attempted loudness and breathing, as well as between different loudness levels during attempted speech for T16 (two-sided Wilcoxon rank-sum test, * $p < 0.05$, 'ns' (not significant) $p > 0.05$; $p_{t15,NORMALLY\text{-}DEEPLY} = 0.71$, $p_{t15,NORMAL\text{-}LOUD} = 0.012$, for all other comparisons in T15 $p = 10^{-10}$, $p_{t16,NORMALLY\text{-}DEEPLY}=0.52$, for all other comparisons in T16 $p = 10^{-10}$). **c.** Firing rates (mean ± s.e.) from example electrodes, computed by averaging across instructed breathing and sentence-loudness trials. Some electrodes exhibited tuning primarily to either the attempted breath task or the speech loudness task. **d.** Accuracy of breath and loudness decoders tested on either same or cross-task conditions. Decoders trained on one task did not outperform chance when evaluated on the other task (one-sided permutation test, * $p < 0.05$, 'ns' (not significant) $p > 0.05$; p = 0.00 wherever *, $p_{t15,breath\text{-}loudness} = 0.47$, $p_{t15,loudness\text{-}breath} = 1$, $p_{t16,breath\text{-}breath} = 0.93$, $p_{t16,breath\text{-}loudness} = 0.42$, $p_{t16,loudness\text{-}breath} = 0.17$). This suggests that neural features associated with attempted loudness and instructed breath are different. Gray circles overlaid on each bar plot show classification accuracy for individual test folds (n = 5 folds). **e.** Confusion matrices for breath and loudness decoders tested on either same-task and or cross-task conditions.

Supplementary Note 1: Loudness encoding does not merely reflect breath depth.
Since modulating loudness levels during speech is often accompanied by changes in breath effort[45], we sought to determine whether the neural features used to decode loudness primarily reflected breath-related signals. We analyzed neural activity during the instructed-breathing (breathe NORMALLY vs. DEEPLY) and sentence-loudness tasks. During these tasks, a breath belt was attached around the participant's chest to record the change in thoracic circumference due to respiration. Breath belt recordings showed that breath belt expansion differed significantly between attempted loudness and instructed breathing for both participants T15 and T16 (two-sided Wilcoxon rank-sum test, $p < 0.05$) (Supp. Fig. 6a-b). However, there was little difference between breathing NORMALLY and DEEPLY. This is consistent with both participants, who have paralysis, reporting difficulty modulating their breath during the instructed-breathing task despite attempting to do so.

We next analyzed neuronal firing rates from both tasks and observed that some electrodes' firing rates modulated much more during the instructed breathing task and others modulated much more during the attempted loudness task (Supp. Fig. 6c), suggesting different neural activity patterns between the two tasks. To quantify this observation, we trained two separate logistic regression decoders using neural features from [-1.5, 1.5] s window around either speech onset or exhalation onset, depending on the task. One decoder was trained to classify breathing as NORMALLY vs. DEEPLY, and the other to classify attempted loudness as NORMAL vs. LOUD. Each model was evaluated both within its respective task and to classify conditions in the other task. For participant T15, within-task decoding accuracy was high: 91% for breath and 82% for loudness, both significantly above chance ($p < 0.05$; permutation test). However, cross-task performance dropped to chance or below-chance levels, indicating that neural representations for attempted breath depth and loudness do not generalize across tasks. Similar results were observed for participant T16, although breath classification accuracy was close to chance even within the instructed-breathing task (Supp. Fig. 6d-e).

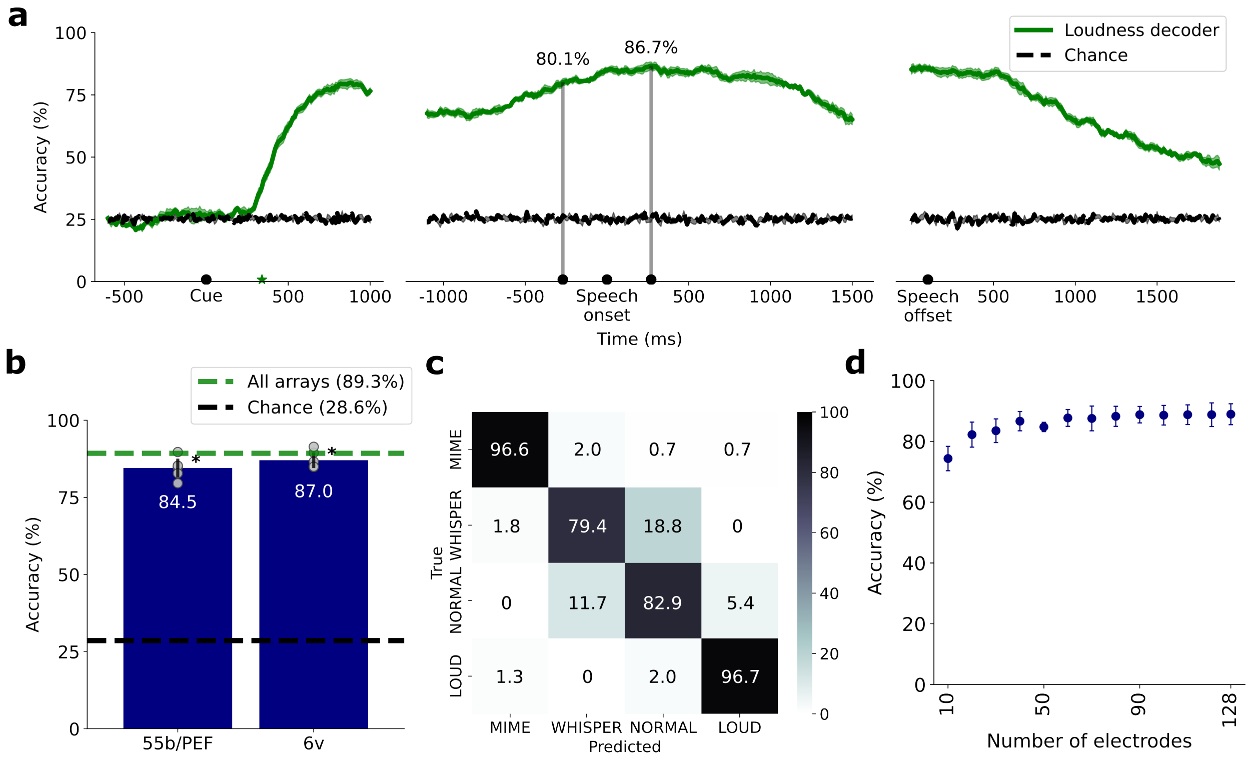

Supplementary Figure 7: T16's loudness could be accurately decoded from neural activity offline. a. Loudness decoders were trained and evaluated on a 400 ms window of neural features with a 10 ms stride. Trial-averaged performance (mean ± s.e.) began to surpass chance at 330 ms after cue onset (green star) and decreased after speech offset (one-sided time-cluster permutation test, $p = 1.9 \times 10^{-2}$). Gray vertical lines mark when decoding accuracy exceeded 80% (270 ms before speech onset) and when maximum accuracy was achieved (270 ms after speech onset). **b.** Classification accuracy (mean ± s.d., n = 6-fold cross-validation) for each array. Performance was significantly above chance for both arrays and is indicated by * (one-sided permutation test, p = 0.00 for each array). Gray circles overlaid on each bar plot show classification accuracy for individual test folds. **c.** Confusion matrix of the decoder's performance using both arrays. Typical confusions were between WHISPER and NORMAL loudness levels. **d.** Classification accuracy (mean ± s.d., n = 10 repeats) when randomly dropping electrodes. Performance was only slightly worse even with the removal of up to half the electrodes.

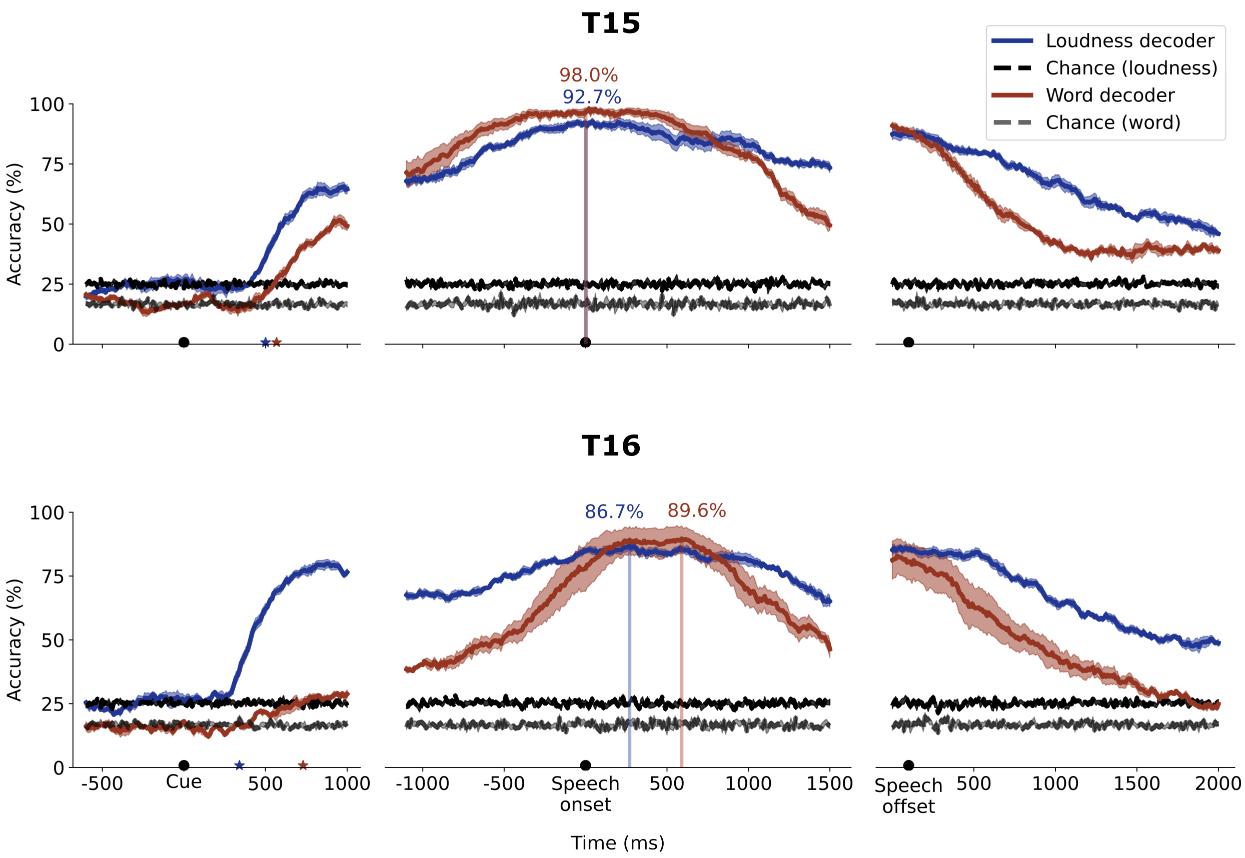

Supplementary Figure 8: Word and loudness decoding across time. Loudness and word decoders were trained and evaluated on every 400 ms window of neural features with 10 ms stride. Trial-averaged loudness decoding performance (mean ± s.e.) significantly surpassed chance at 500 ms and 330 ms after cue onset for T15 and T16, respectively (one-sided time-cluster permutation test, $p_{t15} = 2.9 \times 10^{-2}$, $p_{t16} = 1.9 \times 10^{-2}$, denoted by blue *). Trial-averaged word decoding performance (mean ± s.e.) significantly surpassed chance at 570 ms and 750 ms after cue onset for T15 and T16, respectively (one-sided time-cluster permutation test, $p_{t15} = 5 \times 10^{-3}$, $p_{t16} = 8 \times 10^{-3}$, denoted by red *). Vertical lines mark when peak decoding accuracies were achieved. Both loudness and word decoding accuracy gradually decreased after speech offset, with word decoding accuracy rolling off faster.

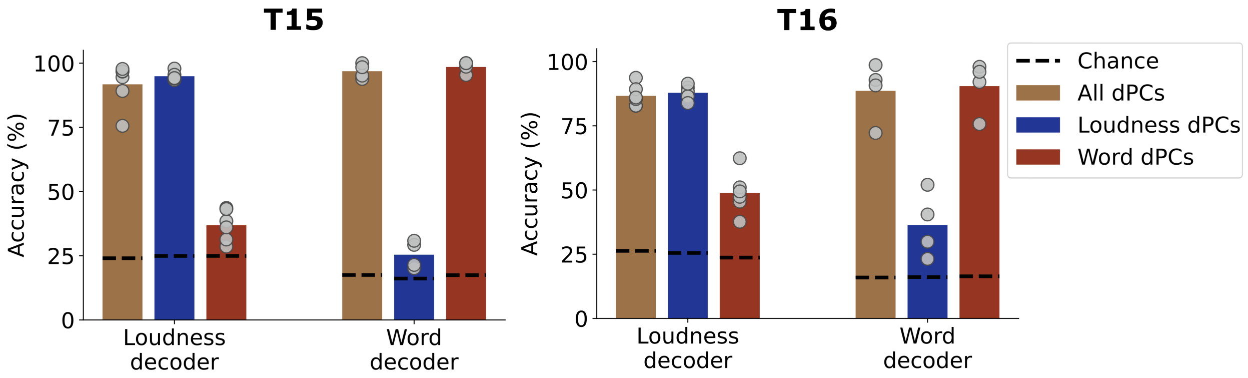

Supplementary Figure 9: Decoding using word- and loudness-specific neural subspaces. For T15 and T16, decoders were trained to predict loudness levels or word identities from either loudness- or word-specific dPC dimensions. Decoders were then tested on neural data projected into either subspace alone or all dPCs. Decoding performance was highest when using either all dPCs or the subspace matched to the task parameter (e.g., decoding words using the word dPCs). Cross-subspace decoding yielded only modestly above-chance performance, indicating low overlap between word- and loudness-specific dPC dimensions. Gray circles overlaid on each bar plot show classification accuracy for individual test folds.

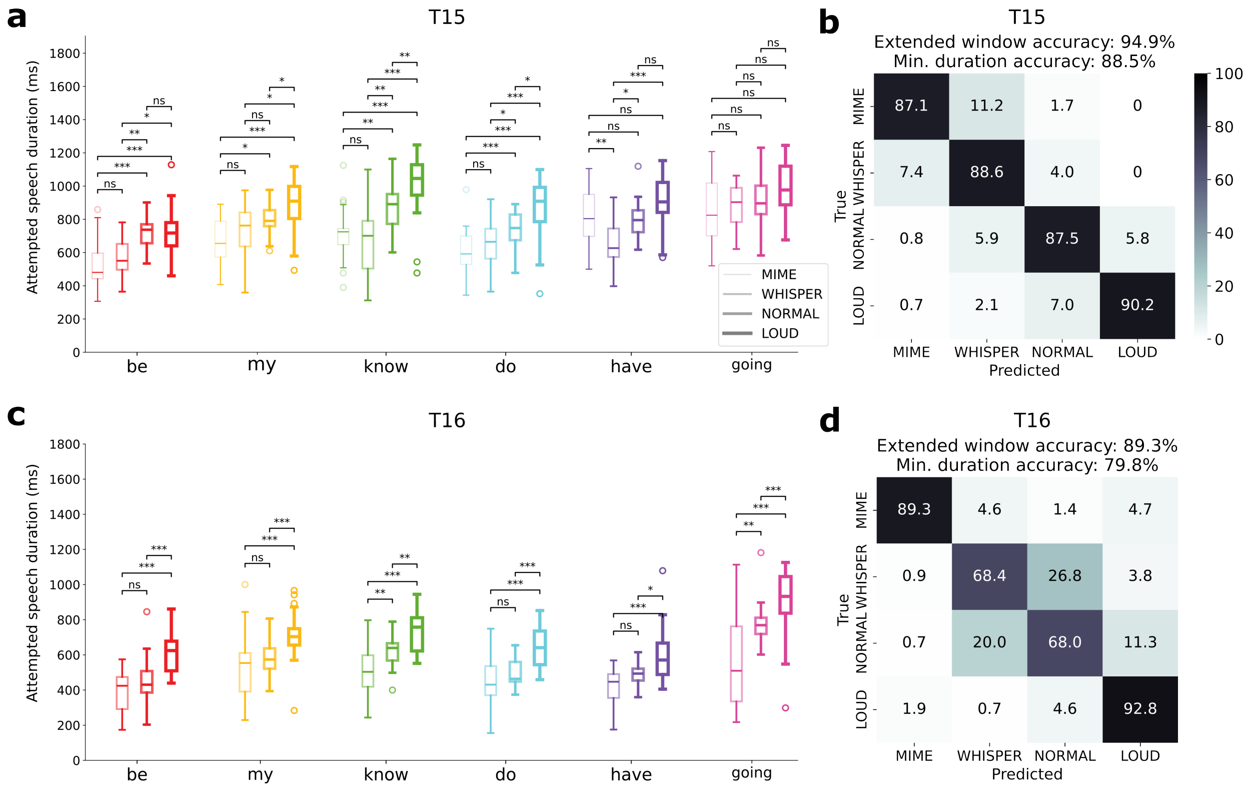

Supplementary Figure 10: Attempted speech duration varied across loudness levels. a, c) Attempted speech duration for T15 and T16, respectively, across different words and loudness levels. Box plots depict the median (center line), 25th and 75th percentiles (lower and upper boundaries of the box), 25th and 75th percentiles ± 1.5 times the interquartile range (whiskers), and outliers (circles). Within each word, speech durations were typically significantly different across loudness levels and increased with louder attempts (two-sided Wilcoxon rank-sum test, * $p < 0.05$, ** $p < 0.01$, *** $p < 0.001$; Bonferroni corrected). **b, d)** Confusion matrix of loudness decoding using neural data from speech onset to the minimum speech duration across conditions (300 ms for T15, 150 ms for T16). The patterns of confusion were consistent with those observed when decoding using the extended [-600, 600] ms neural data window (Fig. 3c, Supp. Fig. 7c). Decoding accuracy remained significantly above chance (which was 25%), with only a 5–10% reduction compared to decoding using the extended window.

Session	Tasks	Dataset details (number of trials)
T15 session #1	Word-loudness<br>(Fig. 1-3, Supp. Fig. 2, 4, 8-10)	135 MIME, 147 WHISPER, 141 NORMAL,<br>143 LOUD
T15 session #2	Sentence-loudness and instructed breath<br>(Supp. Fig. 6)	Loudness: 61 NORMAL, 57 LOUD from 60 trials.<br><br>Breath: 18 NORMALLY, 19 DEEPLY from 8 trials.
T15 session #3	Closed-loop sentence-loudness<br>(Fig. 4)	Train: 200 sentences (first 6 blocks)<br>Evaluation: 50 sentences (last 2 blocks)
T16 session #1	Word loudness<br>(Supp. Fig. 1, 3-5, 7-10)	144 MIME, 128 WHISPER, 149 NORMAL,<br>154 LOUD
T16 session #2	Sentence-loudness and instructed breath<br>(Supp. Fig. 6)	Loudness: 85 NORMAL, 68 LOUD from 85 trials.<br><br>Breath: 50 NORMALLY, 35 DEEPLY from 17 trials.

Supplementary Table 1: Data collection summary.
