# Supplementary material for: Encoding of speech modes and loudness in ventral precentral gyrus
Source: Nat Commun. 2026 Apr 15;17:5301. doi: 10.1038/s41467-026-71284-4 (PMC13270037; doi:10.1038/s41467-026-71284-4)
Supplement: Supplementary file 2 — Description of Additional Supplementary Files [file 41467_2026_71284_MOESM2_ESM.pdf]

Description of additional supplementary files

File name: Supplementary Video 1.mp4

Description: **Closed-loop loudness decoding in a speech BCI.** This video shows 10 consecutive trials of real-time loudness decoding in a brain-to-text BCI, where participant T15 attempted to speak at two loudness levels. The decoded words were capitalized when the loudness decoder predicted *loud* during the word.
