# Supplementary material for: Encoding of speech modes and loudness in ventral precentral gyrus
Source: Nat Commun. 2026 Apr 15;17:5301. doi: 10.1038/s41467-026-71284-4 (PMC13270037; doi:10.1038/s41467-026-71284-4)
Supplement: Supplementary file 19 — Source Data File Captions [file 41467_2026_71284_MOESM19_ESM.docx]

| **Source Data name (as appearing in eProofing)** | **Caption** |
| --- | --- |
| Source Data 1 | Source Data for Figure 1 |
| Source Data 2 | Source Data for Figure 2 |
| Source Data 3 | Source Data for Figure 3 |
| Source Data 4 | Source Data for Supplementary Figure 1 |
| Source Data 5 | Source Data for Supplementary Figure 2 |
| Source Data 6 | Source Data for Supplementary Figure 3 |
| Source Data 7 | Source Data for Supplementary Figure 4 |
| Source Data 8 | Source Data for Supplementary Figure 5 |
| Source Data 9 | Source Data for Supplementary Figure 6 |
| Source Data 10 | Source Data for Supplementary Figure 7 |
| Source Data 11 | Source Data for Supplementary Figure 8 |
| Source Data 12 | Source Data for Supplementary Figure 9 |
| Source Data 13 | Source Data for Supplementary Figure 10 |
