# Supplementary material for: Encoding of speech modes and loudness in ventral precentral gyrus
Source: Nat Commun. 2026 Apr 15;17:5301. doi: 10.1038/s41467-026-71284-4 (PMC13270037; doi:10.1038/s41467-026-71284-4)
Supplement: Supplementary file 5 — Transparent Peer Review file [file 41467_2026_71284_MOESM5_ESM.pdf]

nature portfolio

Peer Review File

Encoding of speech modes and loudness in ventral precentral gyrus

Corresponding Author: Ms Aparna Srinivasan

This file contains all reviewer reports in order by version, followed by all author rebuttals in order by version.

Version 0:

Reviewer comments:

Reviewer #1

(Remarks to the Author)
This work builds upon a foundation of existing high performing Speech-BCI systems in two Speech-BCI users to establish the feasibility of further improving such systems by enabling users to modulate the loudness of produced speech orthogonal to content. This complements previous work (referred to by the authors) in decoding pitch and emphasis. While the step presented hear is incremental and based on only two users, the concept of independently controlling other aspects of speech along with content is crucial to increasing the usability of Speech-BCIs and this work highlights the important role that being able to work with users of functioning BCI systems has in improving such systems.
The methods are sound and the results are clearly presented and support the conclusions.
My only minor concern is that decoding the intention to say something loud or normal can be confounded with other prosodic features of speech, such as pitch and emphasis, as they are effected by a shared set of speech actuators and could potentially all be correlated to increased effort or attention. Thus, future work is needed to establish whether multiple orthogonal aspects of speech in addition to content can be independently controlled simultaneously. While the authors do not make the claim that they can be, I feel that this issue deserves some attention in the limitations and future directions section.

(Remarks on code availability)

Reviewer #2

(Remarks to the Author)
The authors provide evidence for encoding of the speech loudness in vPrG using micro electrode arrays. The manuscript is well written and well organized. This work is the first to show the evidence for loudness encoding on a micro-electrode scale and can be of interest to researchers in BCI applications as well as topics related to human speech production. I think the manuscript is in a good shape as is, however, I have some comments/concerns that hopefully can help the reviewers improve their work:

Some general questions:

- Was there a difference between the duration or rate of speech production between different conditions? Admittedly this is probably hard to investigate in your study but I'd be interested to know your thoughts on that and if/how that can affect the results. Similarly, across different words.

- Was there no auditory feedback across all conditions? are these subjects able to make any sound across different conditions? how does the presence or absence of auditory feedback affect the results? This is especially important since there are some studies showing involvement of PrG in auditory feedback processing (e.g. Ozker M, et al Plos Biology 2022).

- How does the cue affect the results especially the timing of the decoding? Can having a cue make this a little more like a memory task compared to automatic speech?

Minor Edits/comments:

- abstract line 30: "corresponding neural preparatory activity could be detected hundreds of milliseconds before speech onset". I think this can be written in a more precise way. Please highlight the significance of this timing result and maybe consider providing exact timing? I can easily understand that accuracy of the decoding is very high but can't really follow what is significant about the timing? does the fact that it can be detected "hundreds of milliseconds before speech onset" allow you to develop the closed-loop model?

- Introduction line 62-64: Please consider clarifying the relation between naturalistic prosodic features and the speech modes considered in this study. it seems to me that prosodic features have a much finer scale compared to the loud vs mimed for example.

- Figure 1: How does the dynamics of neural activity look across different electrodes within an array? Are the selected electrodes "good" representatives for the entire array? can you perhaps perform some sort of clustering to show representative behavior per region?

- Figure 1: can you show the speech offset on the temporal plots?

- Figure 2: how do the PCs for fig. 2a look like and how are they different with fig. 2b? Are all electrodes from all regions used in the analysis present in Fig2? If so, does the neural ensemble change per array/region or are these dynamics consistent even for only one array?

- Figure 3: It is hard to parse out the timings in Fig3a. Are different time-locked points show different decoders if the data was organized locked to cue, onset, offset? Is there a difference between the duration of the trial given condition and can that affect the timing results? Is there a windowing effect based on the design of the decoder that can effect the timing of the peak decoding performance (ie temporal window of the decoder vs the timings reported here). Can you please discuss/clarify these timings in contrast to what is presented in Fig4, it seems that the decoding in Fig4 has a much sharper temporal curve.

- Overall I would suggest presenting continues temporal with x-ticks at least as supplemental figures.

(Remarks on code availability)
The GitHub link has some analysis code and sample .mat file for one subject. However I was not able to run the codes, the module needed in this line "from session_metadata import incorrect_trials" and "from functions import get_audio_onset_offset" are not included and I don't think these are standard python libraries.

In the code you seem to be referring to "curr_block = int(np.squeeze(data_temp['block_number']))" but block_number is not part of the provided mat file: dict_keys(['__header__', '__version__', '__globals__', 'cue', 'delay_duration_ms', 'threshcross', 'spikepow', 'raw_threshcross', 'predaudio16k'])

I would have loved to be able to plot the firing rates for other significant electrodes but couldn't get the code running; I am not particuraly good with python so this might have been because of my inexperience. However, I suggest you make sure all the required libraries are listed and are easy to access.

Reviewer #3

(Remarks to the Author)
This study examines how speech mode and loudness are encoded in the ventral precentral gyrus and demonstrates their utility for speech brain–computer interfaces. Using intracortical recordings from two participants with paralysis, the authors show that neuronal activity in vPCG is strongly modulated by attempted speech modes (mime, whisper, normal, loud). Loudness could be decoded with high accuracy, including from preparatory activity before speech onset, and integrated into a closed-loop brain-to-text system that modulated text formatting in real time. The work advances current speech neuroprosthesis research by moving beyond decoding what is said to also capture how it is said, enabling more expressive and naturalistic communication.

Overall, the study presents novel and valuable data on how speech loudness and mode are represented in the speech motor cortex, and provides a compelling proof-of-concept for incorporating these expressive features into speech BCI systems.

Major comments:
1. Figure 1 shows firing rates (threshold crossings) modulated by speech loudness, while Figure 2 applies PCA on spike band power. It would be helpful to clarify whether these two neural features capture different or similar information about loudness encoding, and why spike band power was preferred for dimensionality analyses.
2. Relatedly, what do the ensemble coding dynamics look like if threshold crossings are used instead of spike band power?
3. The authors emphasize that loudness could be decoded well before speech onset, indicating preparatory encoding. However, Figures 3 and Supplementary Figure 4 also show that decoding accuracy remains significant for up to ~2 seconds after speech offset in both participants. How should this sustained post-speech decoding be interpreted? Does it reflect residual motor activity, cognitive state signals (e.g., attention or effort), or a delayed return to baseline? A discussion of this

phenomenon would be helpful.
4. In the BCI experiment, the participants performed two tasks simultaneously: modulating loudness while forming words. This may impose additional cognitive or motor demands, compared to the classical speech BCI paradigms. Did this dual-task paradigm affect the performance of the primary brain-to-text decoder, and if so, how was this evaluated?
5. The proportion of variance explained by word (22.7% for T15 vs. 8.1% for T16) and by loudness (13.9% for T15 vs. 32.2% for T16) differs substantially between participants, whereas other dPCA components appear comparable. Can the authors comment on potential reasons for this discrepancy?
6. PCA was performed using [-100, 400] ms around speech onset, while dPCA used [-750, 750] ms. What is the rationale for choosing different analysis windows, and could this influence the observed separability of word vs. loudness encoding?
7. I understand that using threshold crossing facilitates online BCI decoding. But it would be informative to know whether spike sorting was attempted and, if so, how individual neurons encode speech loudness and mode.

Minor:
1. In Figure 1d and Supplementary Figure 1c, a legend could be added to clarify how the size of the black circles corresponds to the number of loudness levels an electrode is tuned to.
2. In Supplementary Figures 3a and 3b, it will be better to use consistent labels, e.g. breathe NORMALLY, breathe DEEPLY, NORMAL loudness level, LOUD loudness level.

(Remarks on code availability)

Reviewer #4

(Remarks to the Author)
In “Encoding of speech modes and loudness in ventral precentral gyrus”, Srinivasan et al. use multiple intracortical electrodes along the precentral gyrus (PCG) to decode speech loudness levels from recorded neural population activity in two dysarthric participants. The work focuses on two main findings: 1) There is separability between the neural representations of loudness levels and words in ventral PCG (vPCG) and 2) loudness levels can be accurately decoded from neural activity in vPCG, specifically in closed-loop brain-computer interface (BCI) contexts. The authors achieve high decoding accuracies of loudness levels across both patients and show that loudness levels are robustly represented across many electrodes in their electrode arrays. The authors conclude that speech modes and loudness are well-represented in the neural activity of vPCG and that future speech BCIs can include this prosodic decoding to provide users with additional expression in their communication.

The authors achieve great success in decoding speech modes and loudness levels from vPCG. Specifically, they show impressive performance of a proof-of-concept speech loudness decoder that aims to extend speech BCIs beyond solely brain-to-text decoding that previous studies have shown. However, I feel that the current work does not sufficiently investigate the encoding of these speech modes and loudness levels in vPCG, as the title suggests. I think the core idea and result here is very interesting, but further work needs to be done to clarify the results/interpretation.

1. While the authors have shown separability between word and loudness subspaces, the abstractness from constraining the analyses to only low-dimensional subspaces inhibits some understanding of how these representations differ. Are there unique spatial patterns associated loudness vs word representations (seems likely as the decomposition is performed on the electrode dimension)? Or certain neuroanatomical locations associated with these separable PCs? The authors could investigate this and provide further interpretability on the separability by associating electrodes in neuroanatomical space to the principal components (e.g. simply visualizing the weights of electrodes for a given PC/dPC associated with loudness or word information). An encoding model predicting spike band power from loudness or word features would provide similarly useful information.

2. Relatedly, the somewhat orthogonal result is quite hard to interpret as presented. There are seemingly two distinct models for how the vPCG encodes loudness: one is that neurons (or populations) that are tuned to a specific word/feature fire work when the loudness increases, the other is that loudness is represented distinctly. It is unclear which of these (or both) are supported with the data given. A clearer result/interpretation would make the manuscript much stronger.

3. The orthogonality analysis in Fig. 2d attempts to quantify separability between dPCs associated with word and loudness, however the conclusion is weakened by ambiguity in that the dPCs are “close to orthogonal”. To strengthen this claim, the authors should consider additional metrics to quantify the lack of overlap between word and loudness representations specifically. Perhaps the authors could compare decoding performance when classifying loudness level or word identity from loudness and word dPCs?

4. Does the decoder have anything to do with the results from the first part of the paper? Or is it simply that he information is present and so it can be decoded online? The manuscript would benefit greatly if there was a stronger link between the first part of the paper (science) and the second (engineering).

5. Please include a label for the colorbar on Fig. 2d. While the upper and lower triangles show slightly different information, a label indicating the overall metric (e.g. “similarity”) would be appreciated.

6. Why are different time windows used for PCA vs dPCA decomposition? My interpretation was that PCA indicated different subspaces for word vs loudness information, and dPCA validated that by explicitly showing different components for word or

loudness information. Using different time windows weakens the use of dPCA as a deeper investigation into the PCA representation.

7. In Fig. 3a, the authors approximately describe timepoints where loudness decoding increased above chance. The authors may consider the use of time-cluster permutation testing to identify temporal regions where loudness decoding significantly differed from chance to more accurately describe the timing of loudness information in vPCG.

8. Please state whether false discovery rate correction was used in cases with multiple significance tests (e.g. Fig. 4b).

(Remarks on code availability)

Reviewer #5

(Remarks to the Author)
I co-reviewed this manuscript with one of the reviewers who provided the listed reports. This is part of the Nature Communications initiative to facilitate training in peer review and to provide appropriate recognition for Early Career Researchers who co-review manuscripts.

(Remarks on code availability)
The github repository is well structured in separating code by different figure analyses and providing instructions on necessary packages for use. However, I was not able to run any of the analysis python files as described in the instructions. There are a few import errors that seem to be due to missing files ("from session_metadata import incorrect trials" or "from functions import get_audio_onset_offset", where session_metadata and functions appear to be non-package files that are not present). This prevented me from running downstream analysis files, as some relied on data saved from earlier files like psth.py. The code provided is useful for determining what analyses were performed and how exactly these analyses were implemented, but fixes should be made for users to run these analyses themselves on the sample data as the README describes.

Version 1:

Reviewer comments:

Reviewer #1

(Remarks to the Author)
The authors have fully addressed my concerns.

Reviewer #2

(Remarks to the Author)
Thank you for addressing all my comments with great detail. I have no further questions or concerns.

-A. Khalilian-Gourtani

Reviewer #3

(Remarks to the Author)
The authors have adequately addressed all my comments and concerns. I appreciate their efforts in improving the clarity of the manuscript.

Signed: Yuanning Li

Reviewer #4

(Remarks to the Author)
We thank the authors for their rigorous work to address our concerns. The authors have greatly clarified the first section of their results related to the encoding of loudness in the vPCG. Specifically, they more strongly show that 1) Speech loudness is largely encoded at the population level, instead of the single-neuron level, and 2) Speech loudness is primarily encoded in distinct population-level neural patterns compared to word identity (though with partial overlap). The additional analyses included in support of point 2 are especially important. The loudness and word decoding analyses from loudness and word dPCs (Response to Reviewers Figure 8) were especially convincing of this point, and we encourage the authors to include it as a supplementary figure in their manuscript. Overall, we feel that the authors have sufficiently addressed our concerns.

Reviewer #5

(Remarks to the Author)
I co-reviewed this manuscript with one of the reviewers who provided the listed reports. This is part of the Nature

Communications initiative to facilitate training in peer review and to provide appropriate recognition for Early Career Researchers who co-review manuscripts.

Open Access This Peer Review File is licensed under a Creative Commons Attribution 4.0 International License, which permits use, sharing, adaptation, distribution and reproduction in any medium or format, as long as you give appropriate credit to the original author(s) and the source, provide a link to the Creative Commons license, and indicate if changes were made.
In cases where reviewers are anonymous, credit should be given to 'Anonymous Referee' and the source.
The images or other third party material in this Peer Review File are included in the article's Creative Commons license, unless indicated otherwise in a credit line to the material. If material is not included in the article's Creative Commons license and your intended use is not permitted by statutory regulation or exceeds the permitted use, you will need to obtain permission directly from the copyright holder.
To view a copy of this license, visit https://creativecommons.org/licenses/by/4.0/

Response to Reviewers

Title: Encoding of speech modes and loudness in ventral precentral gyrus

Manuscript # NCOMMS-25-40410

Dear Editor and Reviewers,

We thank all the reviewers for their insightful and detailed feedback on our manuscript. We appreciate that all reviewers expressed enthusiasm for this study: "...the concept of independently controlling other aspects of speech along with content is crucial to increasing the usability of Speech-BCIs and this work highlights the important role that being able to work with users of functioning BCI systems has in improving such systems." (Reviewer 1); "This work is the first to show the evidence for loudness encoding on a micro-electrode scale and can be of interest to researchers in BCI applications as well as topics related to human speech production." (Reviewer 2); "the study presents novel and valuable data on how speech loudness and mode are represented in the speech motor cortex, and provides a compelling proof-of-concept for incorporating these expressive features into speech BCI systems." (Reviewer 3); "The authors achieve great success in decoding speech modes and loudness levels from vPCG…they show impressive performance of a proof-of-concept speech loudness decoder that aims to extend speech BCIs…" (Reviewer 4).

The reviewers' comments and questions have helped us to make this manuscript stronger and more thoroughly investigate speech modes and loudness encoding in the ventral precentral gyrus. At the beginning of this Response to Reviewers, we first address 3 questions that were shared by multiple reviewers. Later in this document, we address each reviewer's individual comments in detail. Throughout this document, our response to the reviewers is in **blue** text like this. Relevant copied passages from the manuscript are in **black** text, like this. The new text added to the manuscript is in **green**.

Shared questions' responses:

1. Failed code execution (Reviewer #2, Reviewer #5)

Reviewer #2:

> "The GitHub link has some analysis code and sample .mat file for one subject. However I was not able to run the codes…I would have loved to be able to plot the firing rates for other significant electrodes but couldn't get the code running; I am not particularly good with python so this might have been because of my inexperience. However, I suggest you make sure all the required libraries are listed and are easy to access.

Reviewer #5:

“The github repository is well structured in separating code by different figure analyses and providing instructions on necessary packages for use. However, I was not able to run any of the analysis python files as described in the instructions…The code provided is useful for determining what analyses were performed and how exactly these analyses were implemented, but fixes should be made for users to run these analyses themselves on the sample data as the README describes.”

We apologize for the import errors and the issues with code execution. Initially, only sample data were uploaded to the repository. The “analysis” scripts provided there are designed to reproduce our results when run with the full dataset. These scripts (which previously failed to run) now execute correctly using the sample data; however, the results obtained from the sample data are not comparable to those reported in the manuscript. The complete dataset will be uploaded to Dryad upon publication of this manuscript.

We have also added a new folder named **“plotting_scripts”** to the repository. This folder contains Python scripts that read the final analysis outputs (e.g., per-electrode PSTH values for Fig. 1c) and generate the corresponding figures reported in the paper. All scripts within this folder can now be run to reproduce the final analyses and plots presented in the manuscript.

2. Sustained post-speech loudness decoding (Reviewer #2, Reviewer #3)
Reviewer #2:

9. “Figure 3: It is hard to parse out the timings in Fig3a… Can you please discuss/clarify these timings in contrast to what is presented in Fig4, it seems that the decoding in Fig4 has a much sharper temporal curve.”

Reviewer #3:

3. “The authors emphasize that loudness could be decoded well before speech onset, indicating preparatory encoding. However, Figures 3 and Supplementary Figure 4 also show that decoding accuracy remains significant for up to ~2 seconds after speech offset in both participants. How should this sustained post-speech decoding be interpreted? Does it reflect residual motor activity, cognitive state signals (e.g., attention or effort), or a delayed return to baseline? A discussion of this phenomenon would be helpful.”

It is indeed interesting that decoding accuracy remained above chance up to ~2 s after speech offset. Our current data do not allow us to conclusively identify why this is the case, but potential reasons include: some persistent neural activity related to the recent speech action; a more general cognitive state related to something like recent effort (which might, e.g., be higher after loud speech), or – and this is our leading hunch – potential slight differences in the motor plant state at the end of speech (e.g., where the tongue or diaphragm end up) between conditions. While the measurements we have available don’t allow us to answer this, we have added **Supplementary Figure 8** to more prominently show readers this effect, and also contextualize it with a new analysis that shows how word decoding also persists after speech offset (although this word decoding falls off faster). With only 6 word options in these data, this effect would also be consistent with our hypothesis that the participants’ end orofacial posture differs.

Supplementary Figure 8:

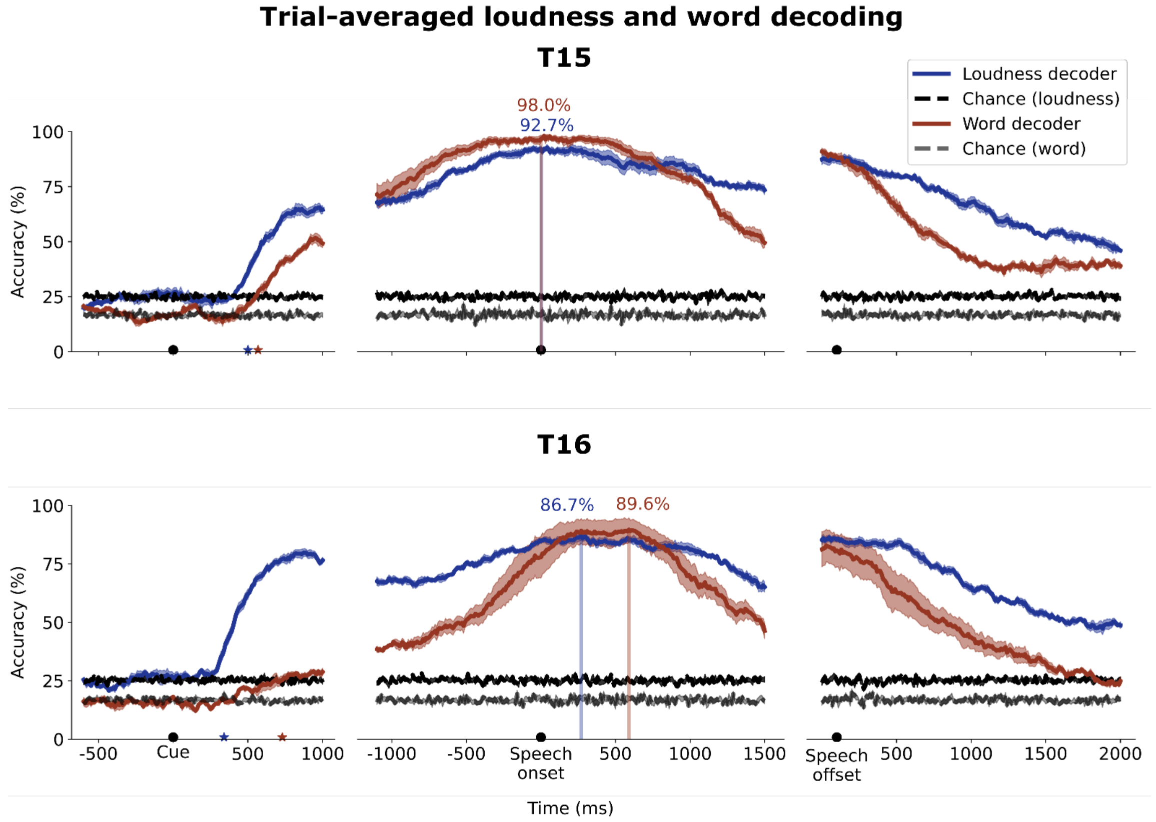

Supplementary Figure 8: Word and loudness decoding across time. Loudness and word decoders were trained and evaluated on every 400 ms window of neural features with 10 ms stride. Loudness decoding performance significantly surpassed chance at 500 ms and 330 ms after cue onset for T15 and T16, respectively (time-cluster permutation test, $p < 0.05$, denoted by blue *). Word decoding performance significantly surpassed chance at 570 ms and 750 ms after cue onset for T15 and T16, respectively (time-cluster permutation test, $p < 0.05$, denoted by red *). Vertical lines mark when peak decoding accuracies were achieved. Both loudness and word decoding accuracy gradually decreased after speech offset, with word decoding accuracy rolling off faster.

We have also briefly remarked upon this effect and a possible explanation in **Results section 2.3, line 190**:

…T16, indicating preparatory encoding of speech loudness. Loudness decoding (and also word decoding) remained significantly above chance for up to ~2s after speech offset in both participants (**Supp. Fig. 8**). The present data do not allow us to resolve the specific cause of this phenomenon, which could reflect the neural correlates of lingering articulatory activity; cognitive-state signals such as recent effort; or potential differences in end-of-speech orofacial and/or diaphragm/body posture across behavioral conditions.

and updated the **Methods section 4.3.5, line 534** with the word decoder training details:

…10 repetitions. For word decoding at every time point (Supp. Fig. 8), we followed a similar process as that for loudness decoding, except the data was split into 4 folds where each fold consisted of trials from a particular loudness level.

3. Different time windows used for PCA and dPCA analysis. (Reviewer # 3, Reviewer # 4)
Reviewer #3:

6. "PCA was performed using [-100, 400] ms around speech onset, while dPCA used [-750, 750] ms. What is the rationale for choosing different analysis windows, and could this influence the observed separability of word vs. loudness encoding?"

Reviewer #4:

6. "Why are different time windows used for PCA vs dPCA decomposition? My interpretation was that PCA indicated different subspaces for word vs loudness information, and dPCA validated that by explicitly showing different components for word or loudness information. Using different time windows weakens the use of dPCA as a deeper investigation into the PCA representation."

We appreciate the reviewers pointing out that the different time windows shown for the PCA and dPCA analyses were distracting / question-provoking. Our original choice of a shorter window for PCA was somewhat arbitrary/aesthetic based on our desire to focus on the neural activity around speech onset where there were prominent differences between conditions (our thinking was that this simple snapshot would then motivate the more comprehensive dPCA analysis). **Response to Reviewers Figure 1** below shows that after expanding the temporal window used for the PCA analysis, the total variance explained by the top 3 PCs is still about the same for both participants, and the relationships between different conditions remain similar [the left-right flip for T15 words' ordering is an arbitrary choice of which angle to view from]. Based on the reviewers' feedback, we have switched to using the same larger [-750, 750] ms window in both analyses for consistency.

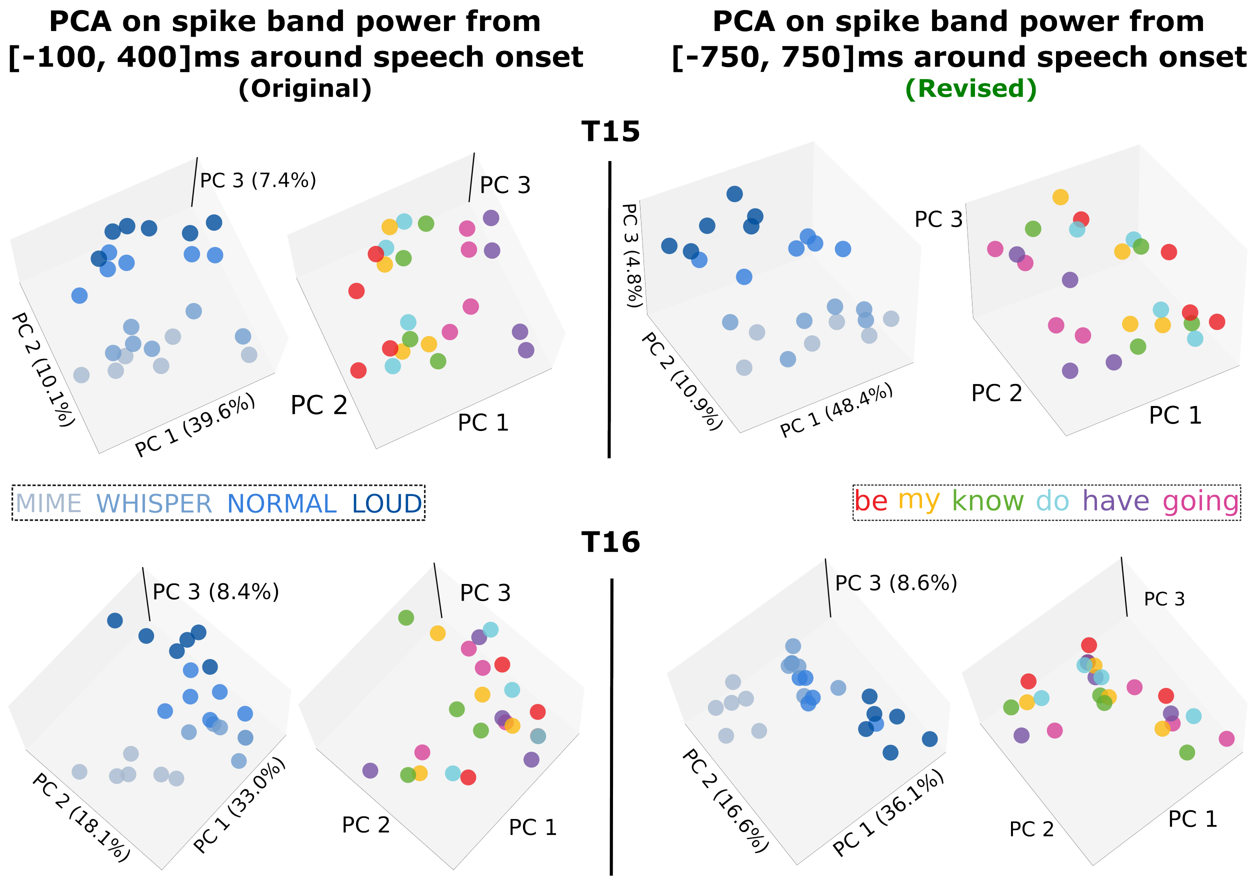

Response to Reviewers Figure 1: PCA on spike band power from two choices of time windows around speech onset.

The updated **Figure 2** and **Supplementary Figure 5** caption now makes clear that the same data are analyzed in both panels:

> **Figure 2. Neural ensemble activity separably encodes loudness from words. a.** PCA projections of trial-averaged spike band power from [-750, 750] ms around speech onset during the word-loudness task…. **b.** dPCA applied to these same data….
>
> **Supplementary Figure 5. T16's neural ensemble activity separably encodes loudness from words. a.** PCA projections of T16's trial-averaged spike band power from [-750, 750] ms around speech onset during the word-loudness task…. **b.** dPCA applied to these same data…

We have also updated **Methods section 4.3.4**, **line 502** to reflect the same:

> For PCA, we analyzed the smoothed spike band power from [-750, 750] ms around speech onset from all electrodes. We obtained the trial-averaged activity for each of the 24 word-loudness conditions (256 electrodes x 150 time bins per condition)....

Individual questions' responses:

Reviewer # 1 (Remarks to the Author)
This work builds upon a foundation of existing high performing Speech-BCI systems in two Speech-BCI users to establish the feasibility of further improving such systems by enabling users to modulate the loudness of produced speech orthogonal to content. This complements previous work (referred to by the authors) in decoding pitch and emphasis. While the step presented here is incremental and based on only two users, the concept of independently controlling other aspects of speech along with content is crucial to increasing the usability of Speech-BCIs and this work highlights the important role that being able to work with users of functioning BCI systems has in improving such systems.

The methods are sound and the results are clearly presented and support the conclusions.
My only minor concern is that decoding the intention to say something loud or normal can be confounded with other prosodic features of speech, such as pitch and emphasis, as they are effected by a shared set of speech actuators and could potentially all be correlated to increased effort or attention. Thus, future work is needed to establish whether multiple orthogonal aspects of speech in addition to content can be independently controlled simultaneously. While the authors do not make the claim that they can be, I feel that this issue deserves some attention in the limitations and future directions section.

We thank the reviewer for their thoughtful and encouraging feedback. We appreciate their recognition of our work as a step toward improving speech BCIs through the independent control of attempted loudness level. We agree with the reviewer that prosodic features such as loudness, pitch, and emphasis often co-vary because they are mediated by overlapping articulatory mechanisms.

We would like to clarify that participants were instructed to *attempt* at different loudness levels according to the condition presented as a cue during a trial. However, given that both participants have dysarthria, it is difficult to determine whether any uninstructed pitch modulations occurred. We have added the following sentence to the **Limitations and future directions section** (**line 329**), emphasizing the need for future work to disentangle these aspects of speech and achieve simultaneous, independent control of multiple prosodic dimensions within speech BCIs.

> …levels of pitch and emphasis modulation in a brain-to-voice BCI. An important next step will be to determine whether multiple prosodic features, such as loudness, pitch, duration, stress, etc., which often covary, are represented in distinct neural dimensions and whether they can be independently controlled in speech BCI applications. The long-term goal of this line of research is to provide continuous and simultaneous control of multiple prosodic features for more natural neural speech synthesis.

Reviewer # 2 (Remarks to the Author):
The authors provide evidence for encoding of the speech loudness in vPrG using micro electrode arrays. The manuscript is well written and well organized. This work is the first to show the evidence for loudness encoding on a micro-electrode scale and can be of interest to researchers in BCI applications as well as topics related to human speech production. I think the manuscript is in a good shape as is, however, I have some comments/concerns that hopefully can help the reviewers improve their work:

We thank the reviewer for their careful assessment of our manuscript. We appreciate their recognition that this work provides the first evidence of loudness encoding at the microelectrode scale and its relevance to both BCI applications and the study of human speech production. We have addressed the reviewer's comments and concerns in detail below.

Some general questions:

1. Was there a difference between the duration or rate of speech production between different conditions? Admittedly this is probably hard to investigate in your study but I'd be interested to know your thoughts on that and if/how that can affect the results. Similarly, across different words.

Yes, we would expect that there would be duration differences since many aspects of speech covary during natural speech, which is an inherent limitation/nuance when studying the neural control of paralinguistic features. We have added a sentence to the **Limitations and future directions** section, **line 329** to point out this limitation:

> …levels of pitch and emphasis modulation in a brain-to-voice BCI. An important next step will be to determine whether multiple prosodic features, such as loudness, pitch, duration, stress, etc., which often covary, are represented in distinct neural dimensions and whether they can be independently controlled in speech BCI applications. The long-term goal of this line of research is to provide continuous and simultaneous control of multiple prosodic features for more natural neural speech synthesis.

Our view is that we can recognize that many elements of speech are intertwined but still make progress in understanding their neural bases by focusing on instructing participants to volitionally modulate specific elements (here, speech mode and loudness), measuring how neural activity changes, and accepting that other elements of speech will covary. We are reassured by the fact that at the end of the day, we are describing the neural correlates of these volitional *behaviors* (which involve a complex set of underlying muscular outputs), rather than trying to isolate e.g., neural control of lung pressure or vocal fold tension.

To quantify and document how durations differed across words and speech modes for these data, we have now analyzed speech duration across words and loudness levels for both participants and report the results in **Supplementary Figure 9** which is copied below.

As described in the Methods, for participant T15 (who does not consistently produce audible speech), speech duration was measured from onsets and offsets extracted from the brain-to-

voice speech BCI output for all loudness conditions. For participant T16, whose dysarthria is less severe, durations for vocalized conditions were measured from the microphone recordings. For T16's MIME condition, speech onset was manually determined from video by noting when she opened her mouth for articulation. However, estimates of T16's speech offset could be unreliable because her mouth did not always return to a closed/rest position after attempted speech. Therefore, T16's MIME condition duration is not reported here, as it could be artificially elevated.

The minimum speech durations across all words and loudness levels were 300 ms for T15 and 150 ms for T16. Speech duration varied across words as expected. Within each word, T15 had significant duration differences between most loudness pairs, except between MIME and WHISPER, and also there was no significant duration difference across loudness levels when he attempted to say “going”. For T16, significant duration differences were typically observed between WHISPER, NORMAL, and LOUD conditions.

To minimize this confound and build confidence that the demonstrated ability to decode loudness was not principally driven by duration differences, we now also show the decoding performance (and confusion matrix) obtained when classifying loudness levels using only neural data from speech onset to the earliest speech offset time across conditions (i.e., the analysis window only captured speaking epochs for all conditions). For T15, decoding accuracy using the original [-600, 600] ms neural window was 94%, and using only this minimum speech duration window of [0, 300] ms it was 88%. Similarly, for T16, decoding accuracy using the original window was 89%, and using the minimum duration window of [0, 150] ms it was 79%. While there is a small drop in performance with the reduced window (as one would expect from reducing the duration of data used for decoding), similar patterns of confusion were observed, and decoding remained highly above chance.

We have added the following details to the manuscript to address variations in attempted speech duration:

Supplementary Figure 9:

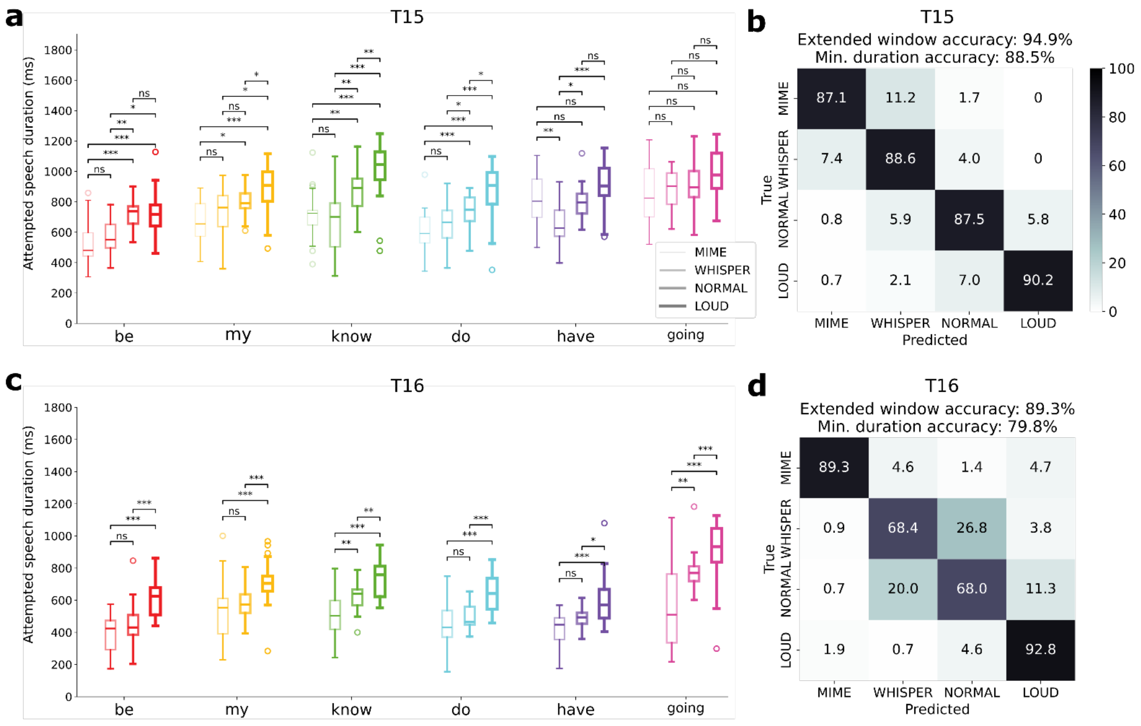

Supplementary Figure 9: Attempted speech duration varied across loudness levels. a, c) Attempted speech duration for T15 and T16, respectively, across different words and loudness levels. Within each word, speech durations were typically significantly different across loudness levels and increased with louder attempts. (Wilcoxon rank-sum test, * $p < 0.05$, ** $p < 0.01$, *** $p < 0.001$; Bonferroni corrected). **b, d)** Confusion matrix of loudness decoding using neural data from speech onset to the minimum speech duration across conditions (300 ms for T15, 150 ms for T16). The patterns of confusion were consistent with those observed when decoding using the extended [-600, 600] ms neural data window (Fig. 3c, Supp. Fig. 7c). Decoding accuracy remained significantly above chance (which was 25%), with only a 5–10% reduction compared to decoding using the extended window.

Results section 2.3, line 223:

Although the participants were only instructed to vary their speech mode and loudness, we expected that other elements of prosody would inevitably covary, including speech duration. To minimize the potential confounding effects that loudness-related differences in speech duration might have contributed to decoding, we also performed decoding using only neural data from speech onset to each participant's across-condition shortest speech duration (300 ms for T15, 150 ms for T16; **Supp. Fig. 9**). Decoding accuracy decreased only 5% for T15 and 10% for T16, indicating that loudness could still be accurately decoded when minimizing potential contributions from speech duration–related neural correlates.

2. Was there no auditory feedback across all conditions? are these subjects able to make any sound across different conditions? how does the presence or absence of auditory feedback affect the results? This is especially important since there are some studies showing involvement of PrG in auditory feedback processing (e.g. Ozker M, et al Plos Biology 2022).

We thank the reviewer for asking about auditory feedback. Both participants attempted to vocalize across all conditions except MIME and WHISPER, but due to their dysarthria the vocal output was greatly reduced. We now report this information in the Methods.

Methods section 4.2.1, line 376:

> These speech modes and loudness levels were chosen such that they were subjectively discernible from one another for each participant. This was confirmed by having the participants tell us that they found these conditions intuitive. Both participants were able to perform the task, although their vocal output was reduced due to their dysarthria – especially for T15.

Notably, we observed robust neural differences between loudness levels even before voice onset, indicating that these effects are not solely driven by auditory feedback. As such, our study likely underestimates any potential contribution of feedback-mediated neural activity that would be stronger in able speakers. We have added a discussion of this point in the Limitations and future directions section of the revised manuscript (**Limitations and future directions, line 314**; note that the added reference 34 is the Ozker et al. study you suggested).

> Different speech modes and loudness levels could produce distinct auditory feedback, which could influence motor cortical activity[34]. In our case participants attempted to vocalize in all conditions except MIME and WHISPER, but their vocal output was substantially reduced due to dysarthria, particularly for T15. As a result, our study likely underestimates the potential contribution of feedback-mediated neural differences across conditions that would be present in healthy speakers. Furthermore, we observed robust neural differences between loudness conditions even before voice onset, suggesting that the reported effects are not strongly dependent on auditory feedback.

3. How does the cue affect the results, especially the timing of the decoding? Can having a cue make this a little more like a memory task compared to automatic speech?

We apologize if the manuscript was unclear regarding the task design. The prompt was displayed on-screen throughout each trial, meaning there was no memory component to the behavior. We have added this sentence to the Methods section to make it clear.

Methods section 4.2, line 365:

> …the participants ended the trials using an eye tracker after attempting the cue. The cue was present for the entire duration of a trial. ….

Our analyses (Figures 1 and 3) are aligned both to the cue and to speech onset. This dual alignment allows us to examine neural differences while minimizing the influence of potential reaction time variability. Similarly, the dPCA analyses (Figure 2) are aligned to speech onset to focus on neural differences related to producing different speech modes rather than responses to the cue itself or reaction time-related differences.

We also note that the use of single words for most of the study (except the final closed-loop sentence task) reduces memory and cognitive demands, helping to isolate the neural encoding of speech-motor differences which we are studying here. Studying differences across speech modes during more naturalistic, self-directed (uncued) speech is an interesting direction for future work. However, such paradigms introduce substantial complexity, including uncontrolled cognitive (language) processes whose timing cannot be precisely measured, and the inability to trial-average across behaviorally similar repetitions. For the purpose of this study, we therefore focused on a prompted task.

Minor Edits/comments:

4. abstract line 30: "corresponding neural preparatory activity could be detected hundreds of milliseconds before speech onset". I think this can be written in a more precise way. Please highlight the significance of this timing result and maybe consider providing exact timing? I can easily understand that accuracy of the decoding is very high but can't really follow what is significant about the timing? Does the fact that it can be detected "hundreds of milliseconds before speech onset" allow you to develop the closed-loop model?

We appreciate this feedback that we could be more specific while still staying succinct in the Abstract. We have now revised the abstract to include these exact timing values to make the statement more precise.

Abstract, line 29:

> Attempted mode/loudness could be decoded from vPCG with 94% and 89% accuracy for the two participants, and corresponding neural preparatory activity at 640 ms and 270 ms before speech onset enabled 80% decoding accuracy, respectively.

Due to the limited space in the abstract to elaborate, we have instead added the following sentence to the Discussion to expand upon the significance of the timing.

Discussion, line 291:

> The presence of preparatory speech loudness- and mode-related activity prior to speech onset further supports vPCG's role in volitional control of attempted loudness and prosody more broadly. Such preparatory encoding could also facilitate low-latency online decoding such as instantaneous prosodic modulation of synthesized voice[21].

5. Introduction line 62-64: Please consider clarifying the relation between naturalistic prosodic features and the speech modes considered in this study. It seems to me that prosodic features have a much finer scale compared to the loud vs mimed for example.

We thank the reviewer for pointing out that speech loudness is merely one element of prosody, and that speaking whole words in different modes and loudness levels as was done here is a coarser (and slower-varying) voice modulation than the finer-scaled modulation often present in naturalistic speech. We have updated the following lines in the Introduction to reflect this.

Introduction, line 63:

> Recent BCI research has demonstrated that the phonemic content of attempted overt[20–23] or mimed speech[26–28] can be decoded from the speech motor cortex into text or voice output. However, the ability to modulate speech BCI output with sustained prosodic modulations, such as changing words' loudness, has not been previously demonstrated.

We have also noted this limitation in the updated Limitations and future directions section.

Limitations and future directions, line 322:

> This study examined loudness and speech mode modulation at the relatively coarse timescale of whole words, and our closed-loop proof-of-concept loudness decoder was limited to binary classification (normal vs. loud speech) for modulating the text formatting in a BCI. Future work should explore more rapidly varying continuous control of loudness and speech mode.

6. Figure 1: How does the dynamics of neural activity look across different electrodes within an array? Are the selected electrodes "good" representatives for the entire array? can you perhaps perform some sort of clustering to show representative behavior per region?

We agree that after seeing the four example electrodes' PSTHs, readers would naturally be curious as to what other electrodes show. To provide a comprehensive view of these unique human intracortical data, we have added **Supplementary Figure 2** showing the firing rates per electrode for participant T15 (and **Supplementary Figure 3** for participant T16). Across all electrodes, we observe a wide variety of firing rate modulations for the different loudness conditions. For example, many electrodes have firing rates increasing for all loudness levels around speech onset and then decreasing around speech offset. Some electrodes show selective tuning for a particular loudness level. There are also electrodes for which the neuronal activity did not modulate much compared to the baseline activity around the beginning of the trial.

Here are the supplementary figures (note that these will be very high resolution in the revised manuscript so readers can zoom in to see each electrode clearly).

Supplementary Figure 2:

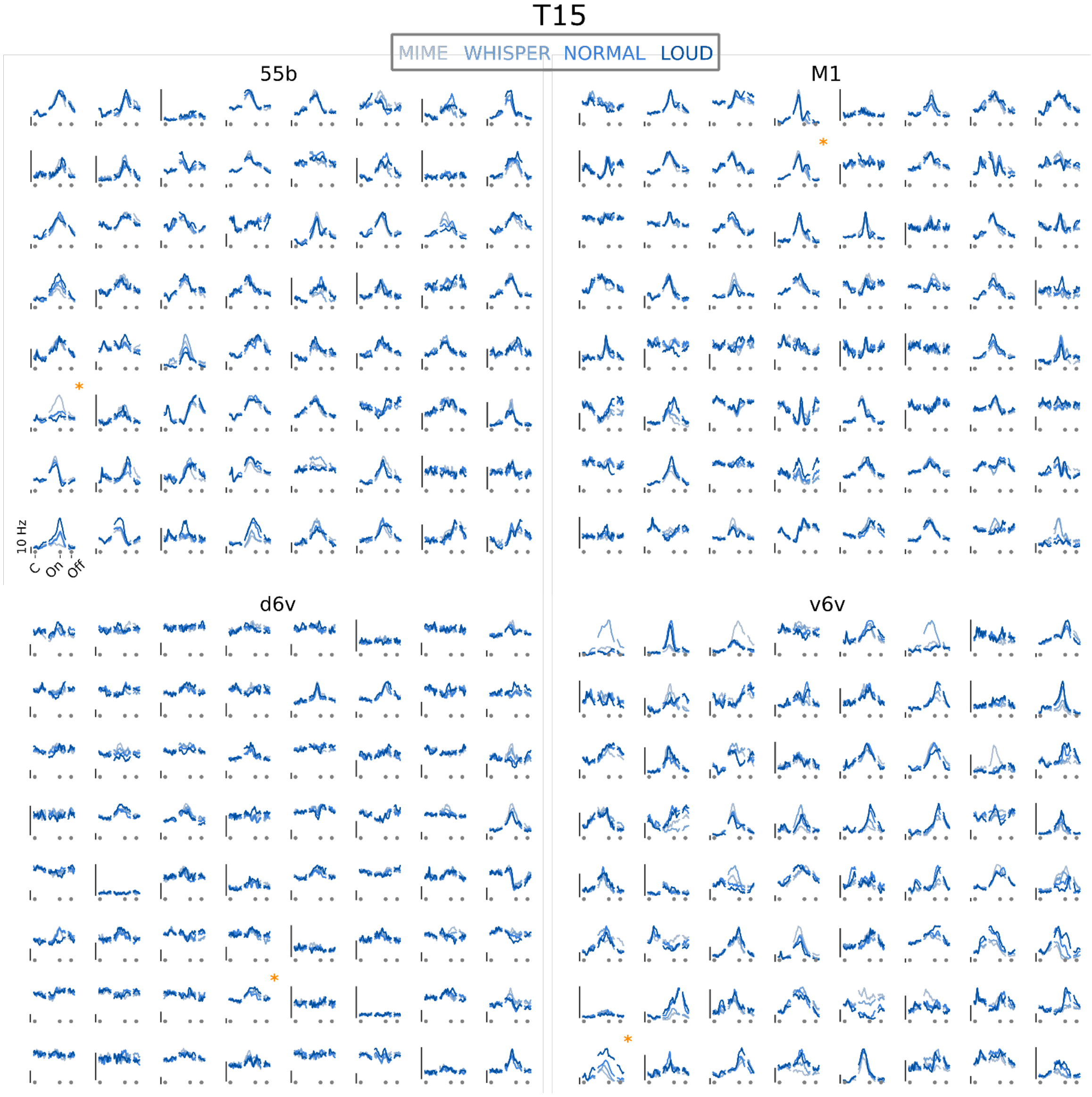

Supplementary Figure 2. All electrodes' firing rates for T15. Firing rates per electrode were computed by trial-averaging within each loudness condition. Neural activity was aligned to cue onset ('C', left), speech onset ('On', middle), and speech offset ('Off', right). Electrodes exhibit different patterns of modulation across vPCG. The four electrodes shown as examples in Fig. 1 are marked with gold stars.

Supplementary Figure 3:

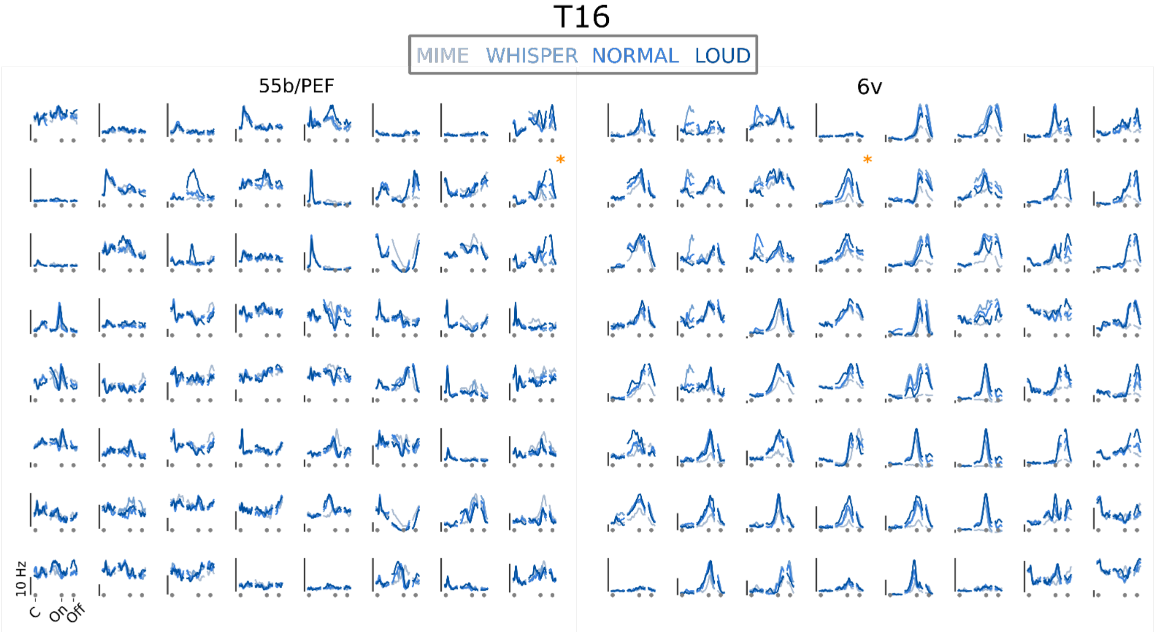

Supplementary Figure 3. All electrodes' firing rates for T16. Firing rates per electrode were computed by trial-averaging within each loudness condition. Neural activity was aligned to cue onset ('C', left), speech onset ('On', middle), and speech offset ('Off', right). Electrodes exhibit different patterns of modulation across vPCG. The two electrodes shown as examples in Supp. Fig. 1 are marked with gold stars.

We refer to these new figures in **Results section 2.1, line 109** as follows:

…Firing rate activity for all electrodes is shown in **Supp. Fig. 2** for T15 and **Supp. Fig. 3** for T16.

7. Figure 1: can you show the speech offset on the temporal plots?

Absolutely. All figures with temporal plots now show speech offsets too (for example in **Fig. 1, Supp. Fig. 1, Supp. Fig. 2-3**). One example of this change (**Fig. 1c**) is copied below:

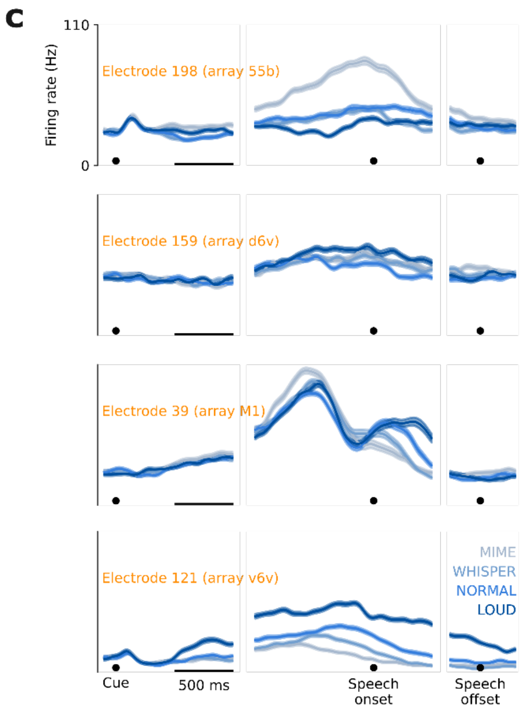

8. Figure 2: how do the PCs for fig. 2a look like and how are they different with fig. 2b? Are all electrodes from all regions used in the analysis present in Fig2? If so, does the neural ensemble change per array/region or are these dynamics consistent even for only one array?

Fig. 2a shows the trial-averaged and time-averaged PCA projections using the top-3 PCs (i.e., unsupervised dimensionality reduction that just maximizes neural variance). Fig. 2b shows the trial-averaged projections from dPCs (demixed principal components, a targeted dimensionality reduction technique that maximizes neural variance related to different task parameters). We would like to clarify that both PCA and dPCA were performed on all the electrodes. In order to make this clearer, we have modified the sentence in the Methods section as follows.

Methods section 4.3.4, line 503 and line 509:

> For PCA, we analyzed the smoothed spike band power from [-750, 750] ms around speech onset from all electrodes…. For the dPCA analysis, which also reduced dimensionality across electrodes, we used the smoothed spike band power from [-750, 750] ms around speech onset from all electrodes and decomposed the neural activity into 20 components…

Re: how the PCs differ from dPCs, we first measured the subspace angle between the top-3 PC basis vectors and the top-3 dPC basis vectors. Principal angles between the top-3 PCA and top-3 dPCA axes were 22.2°, 22.1° and 16.3° (cosines 0.93, 0.93, 0.96), indicating substantial overlap between the two subspaces. In other words, the dominant population axes discovered by PCA largely coincide with the task-demixed axes recovered by dPCA. We have added text to the

Methods to better explain the relationship between PCA and dPCA and why we used each analysis.

Methods section 4.3.4, line 490:

> To determine if the ensemble neural activity encoded attempted loudness, we performed PCA and demixed PCA[40] on the word-loudness task data (Fig. 2, Supp. Fig. 5). Here PCA was used as a standard unsupervised data visualization technique to show the across-electrodes patterns that captured the most variance. dPCA was used as a more detailed and hypothesis-driven targeted dimensionality reduction technique to isolate variance related to different task parameters[40]. …

We also quantified the percent weight contributed by each array for the top-3 PCs and top-3 dPCs (**Response to Reviewers Figure 2**). In T15, the PCA basis vectors showed the highest contribution from v6v, whereas the dPCA basis vectors (decoder matrix values) were larger in 55b. This difference likely reflects the fact that PCA captures overall neural variance (including task-unspecific fluctuations), while dPCA isolates task-modulated variance; thus, dPCA may emphasize electrodes in 55b that carry stronger word- or loudness-related signals even if v6v exhibits greater overall variability. In both PCA and dPCA, d6v had the lowest contribution, consistent with the relatively weak word and loudness encoding observed in that array (Fig. 1d–e). In T16, we observed a similar pattern with both PCA and dPCA – greater weights were attributed to 6v which shows strong encoding to both word and loudness (Supp. Fig. 5c–d).

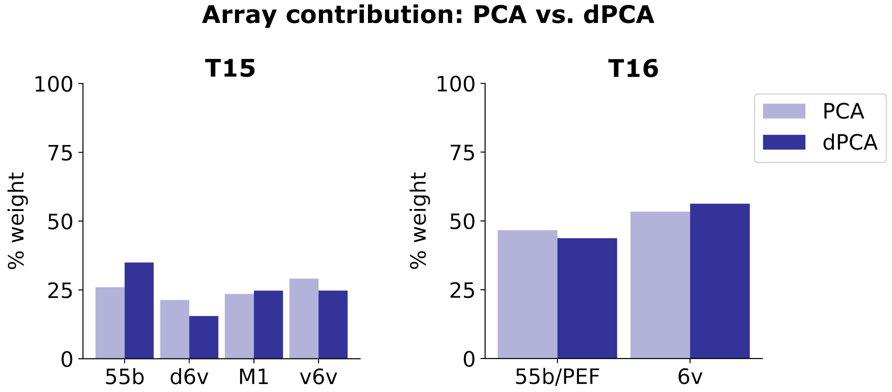

Response to Reviewers Figure 2: Array contribution in PCA and dPCA. Here the weights add up to 100%, i.e., this is the fraction of the top 3 PCs (or top 15 dPCs) contributed by each array.

Since these analyses did not reveal any striking or surprising patterns in either the relationship between the PCs and dPCs or the array contribution, we feel that adding these results to the revised manuscript would be too detailed / add clutter. However, we have added a brief note to the Methods to state that all arrays contributed to the reduced-dimension data projections.

Methods section 4.3.4, line 513:

> For plotting purposes, we chose the first 15 dPCA components. Empirically, all four (T15) or two (T16) arrays contributed substantially to the PCA and dPCA loadings (not shown).

Re: whether the neural ensemble dynamics change across arrays, we performed dPCA on each array separately. For T15 (**Response to Reviewers Figure 3**), we find that the neural dynamics indeed differ somewhat across arrays (which we'd expect, since the dynamics across all four arrays are a composite of what's happening on each array). For example, the separation patterns between words in each array's individual top word-dPC show different patterns. Similarly, the loudness-related separation in the top loudness-dPC also varies (but note that there are other word- or loudness- dPCs for both the all-arrays and individual-array dimensionality reductions, and that comparing specific rank-ordered dPCs is prone to exaggerating differences that may just reflect a switch in e.g. largest and 2nd-largest dPC of a particular condition). Nonetheless, the fraction of explained variance across task-related dPCs follows a similar trend in all arrays: condition-invariant variance dominates, followed by word-related and then loudness-related variance – except in v6v, where loudness is more strongly encoded than words. These findings are consistent with the relative strength of loudness and word encoding reported in Fig. 1d–e. For T16 (**Response to Reviewers Figure 4**), we also observe some differences between the two arrays. In 55b/PEF, there is little visible separation between different words in the top word-dPC projection (also very low explained variance), consistent with Supp. Fig. 1d where word encoding in 55b is weak. Overall, T16's 6v shows stronger loudness and word representations compared to 55b. Since these array-specific dPCA differences do not alter the overall conclusions of the manuscript and are also captured in the aforementioned other analyses, we prefer to include these array-specific dPCA figures only in the Response to Reviewers document unless the reviewer or editor feels strongly otherwise.

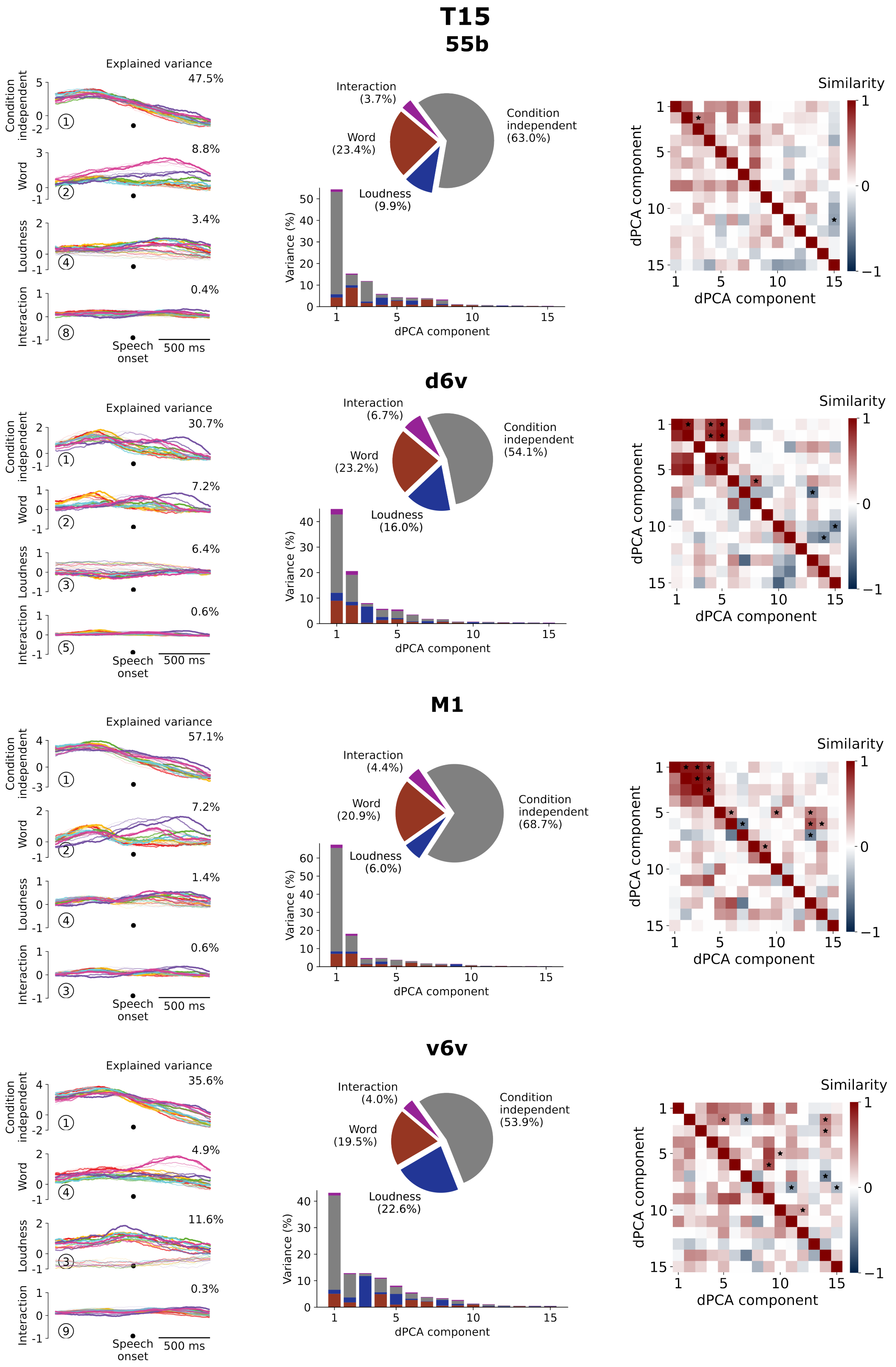

Response to Reviewers Figure 3: dPCA on each array for T15. In the dPC projections, each plot has 24 curves (4 loudness levels × 6 words), with loudness represented by increasing saturation and linewidth from MIME up to LOUD, and words shown in different colors.

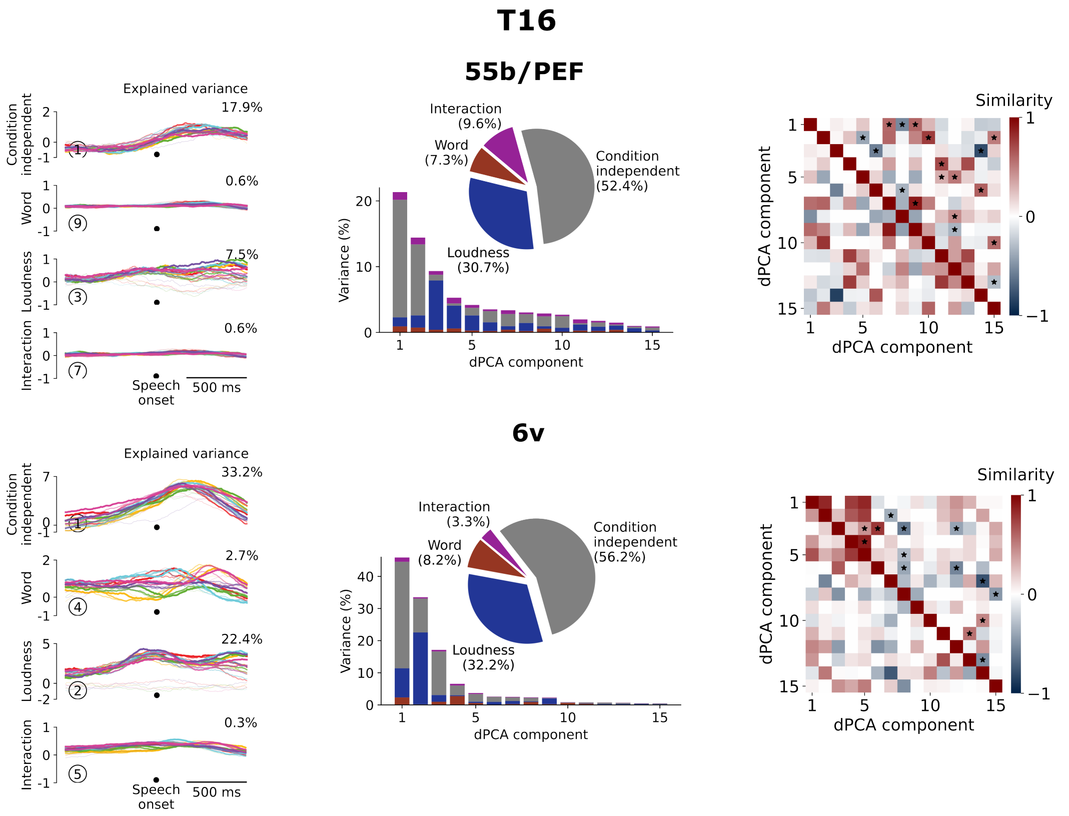

Response to Reviewers Figure 4: dPCA on each array for T16. In the dPC projections, each plot has 24 curves (4 loudness levels × 6 words), with loudness represented by increasing saturation and linewidth from MIME up to LOUD, and words shown in different colors.

9. Figure 3: It is hard to parse out the timings in Fig3a. Are different time-locked points show different decoders if the data was organized locked to cue, onset, offset? Is there a difference between the duration of the trial given condition and can that affect the timing results? Is there a windowing effect based on the design of the decoder that can effect the timing of the peak decoding performance (ie temporal window of the decoder vs the timings reported here). Can you please discuss/clarify these timings in contrast to what is presented in Fig4, it seems that the decoding in Fig4 has a much sharper temporal curve.

We thank the reviewer for these questions, which indicate to us that we need to more clearly unpack Figure 3's key methods for the reader. We address each one of your questions below.

Re: time-locked points and decoding; In Fig. 3a, we show the decoding accuracies by aligning the trials to different events namely, cue onset, speech onset and speech offset. For each time point (= 10ms) in this plot, we trained its own loudness decoder on 400 ms of neural features preceding that time point and plotted the decoding accuracy (in other words, we're asking the question, "How much information is present in this time window", as opposed to, "how well would

a fixed decoder work at every phase of the trial). We apologize that this wasn't clear. We have updated the Methods to reflect this.

Methods section 4.3.5, line 519:

> For offline loudness decoding (Fig. 3, Supp. Fig. 7), we trained multinomial logistic regression models on smoothed neural features from the word-loudness task to predict the four loudness levels. A separate loudness decoder was trained and applied to 400 ms windows of neural features with a 10 ms stride (Fig. 3a, Supp. Fig. 7a). The decoding accuracy at every time point shown in the plots was obtained from a loudness decoder evaluated on the past 400 ms of neural features.

Re: trial duration and timing of the results; all trials were of the same duration with a 2 s delay period and 3 or 4 s go-period. By aligning the trials to different events such as cue (prompt) and speech onset, we are able to determine the amount of loudness-related information in different time windows with respect to these events. This is critical as the reaction time for each participant varies and they may not attempt to speak immediately after the 'go' cue. As with any trial-averaged analysis, the "crispness" of the alignment (due to trial-to-trial behavioral variability) will degrade the farther a time point is from the alignment event, which is another reason we used multiple alignment events.

Re: windowing effect; to measure the windowing effect, we repeated the analysis where decoding accuracy at every 10 ms time bin is obtained from a loudness decoder trained on the past 100 ms (instead of 400 ms) of neural features (**Response to Reviewers Figure 5**). For participant T15, we observed that there was a small drop in peak decoding accuracy from 92.7% to 90.2% (not surprising given that the decoder was provided with less data), but this peak was still achieved at speech onset. For T16, peak decoding accuracy dropped slightly to 82.7% from 86.7%. Further the peak decoding accuracy was now achieved at speech onset instead of 270 ms after speech onset. Therefore, reducing the decoder window length led to a 2–4% drop in peak decoding performance, likely because the shorter window captures less temporal context and thus reduces the decoder's ability to integrate slower neural modulations related to loudness. The shift in the timing of peak performance toward speech onset can be explained by the reduced temporal overlap between consecutive windows, but seems to affect the decoding performance for one of the participants. The decoding accuracy decreases faster for the decoder with the 100ms window (as expected), however in both cases the decoding accuracy continues to be above chance after speech offset. Given that the overall conclusions remain unchanged using a 100 ms versus a 400 ms sliding window, we have opted not to include this additional figure as a supplemental figure (the manuscript has already gained a number of supplemental figures) - but if the reviewer or editor feel strongly this should be included, we're happy to do so.

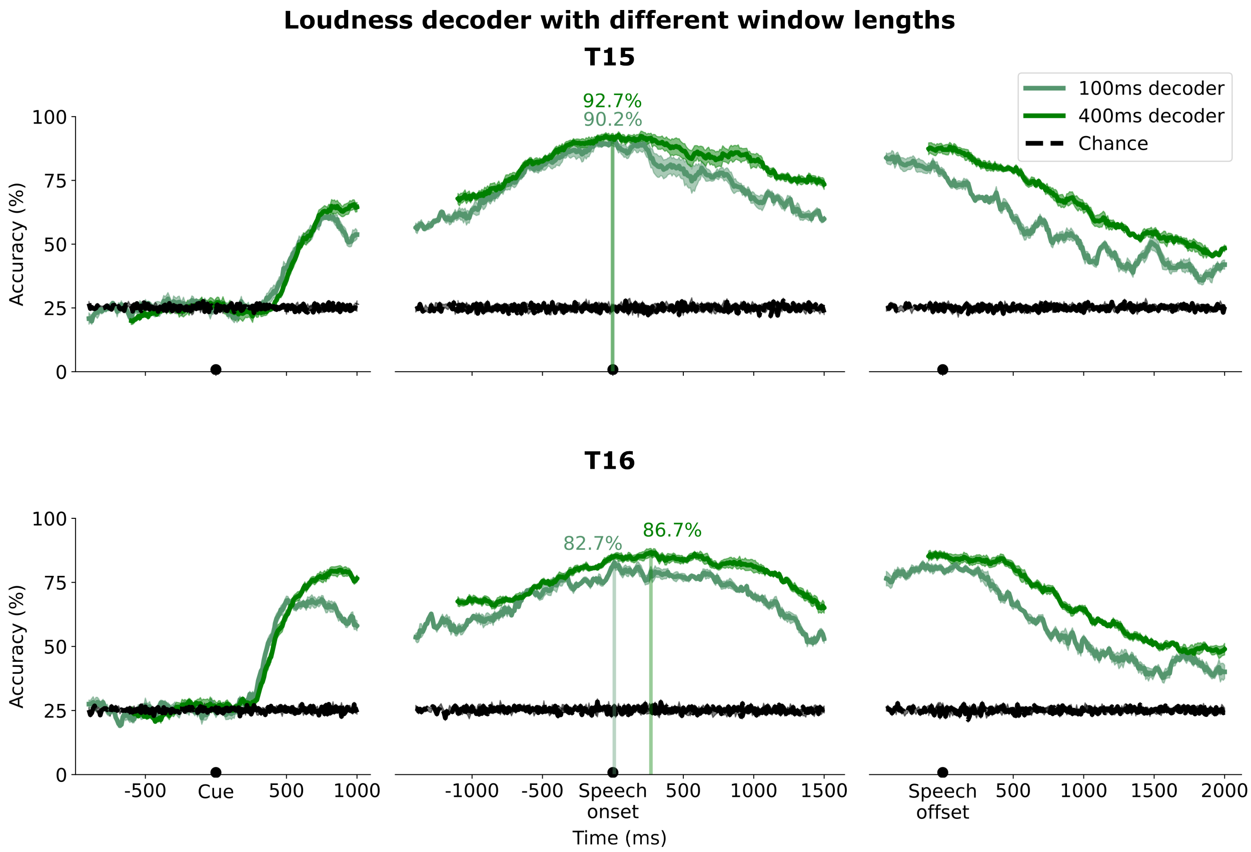

Response to Reviewers Figure 5: Comparison of decoding loudness using decoders of different window length (100 vs. 400 ms). There is a small drop in performance when using a shorter window of neural data for decoding.

Re: comparison to sharper temporal curve observed in Fig. 4; We would like to clarify that Fig. 4c shows the loudness decoder outputs for a single example trial, whereas Fig. 3a depicts the trial-averaged decoding accuracy across time. Furthermore, in Fig. 4c, the loudness decoder was trained to predict an additional “silence” class in addition to the two loudness levels, as described in the Methods. Consequently, it can explicitly classify periods when the participant is not attempting to speak, resulting in a sharper drop-off and higher apparent decoding accuracy. In contrast, in Fig. 3a, at each time point a separate decoder was trained on the past 400 ms of neural features to predict among four loudness levels. The slower decline in decoding accuracy after speech offset in this analysis may reflect residual neural activity due to orofacial postures. This has been further addressed in **Shared Question #2** above. We have updated **Figure 3** and **Supplementary Figure 7 caption** to make it clear that these are trial-averaged results.

> **Figure 3: Loudness could be accurately decoded from neural activity offline. a.** Loudness decoders were trained and evaluated on a 400 ms window of neural features with a 10 ms stride. Trial-averaged performance began to surpass chance at 500 ms after cue onset (green star) and decreased after speech offset (time-cluster permutation test, $p < 0.05$).

Supplementary Figure 7: T16's loudness could be accurately decoded from neural activity offline. a. Loudness decoders were trained and evaluated on a 400 ms window of neural features with a 10 ms stride. Trial-averaged performance began to surpass chance at 330 ms after cue onset (green star) and decreased after speech offset (time-cluster permutation test, $p < 0.05$).

10. Overall I would suggest presenting continuous temporal with x-ticks at least as supplemental figures.

While we initially chose a minimalist style without x-ticks to focus the eye on the data (high "data ink ratio" to use the graphic design term), we understand that preferences vary. As a compromise, we have added x-ticks to most of the supplemental figures for readers who prefer a more conventional temporal axis.

Reviewer #2 (Remarks on code availability):

The GitHub link has some analysis code and sample .mat file for one subject. However I was not able to run the codes, the module needed in this line "from session_metadata import incorrect_trials" and "from functions import get_audio_onset_offset" are not included and I don't think these are standard python libraries.

In the code you seem to be referring to "curr_block = int(np.squeeze(data_temp['block_number']))" but block_number is not part of the provided mat file: dict_keys(['__header__', '__version__', '__globals__', 'cue', 'delay_duration_ms', 'threshcross', 'spikepow', 'raw_threshcross', 'predaudio16k'])

I would have loved to be able to plot the firing rates for other significant electrodes but couldn't get the code running; I am not particularly good with python so this might have been because of my inexperience. However, I suggest you make sure all the required libraries are listed and are easy to access.

Thank you for testing the code and we apologize for the errors; we have addressed this in **Shared Question #1** near the top of this document.

Reviewer #3 (Remarks to the Author):

This study examines how speech mode and loudness are encoded in the ventral precentral gyrus and demonstrates their utility for speech brain–computer interfaces. Using intracortical recordings from two participants with paralysis, the authors show that neuronal activity in vPCG is strongly modulated by attempted speech modes (mime, whisper, normal, loud). Loudness could be decoded with high accuracy, including from preparatory activity before speech onset, and

integrated into a closed-loop brain-to-text system that modulated text formatting in real time. The work advances current speech neuroprosthesis research by moving beyond decoding what is said to also capture how it is said, enabling more expressive and naturalistic communication.

Overall, the study presents novel and valuable data on how speech loudness and mode are represented in the speech motor cortex, and provides a compelling proof-of-concept for incorporating these expressive features into speech BCI systems.

We appreciate the reviewer's encouraging comments, their assessment that this work contributes to understanding neural encoding of speech mode and loudness, and has potential to enhance expressive communication in speech BCI systems.

Major comments:

1. Figure 1 shows firing rates (threshold crossings) modulated by speech loudness, while Figure 2 applies PCA on spike band power. It would be helpful to clarify whether these two neural features capture different or similar information about loudness encoding, and why spike band power was preferred for dimensionality analyses.

In our data , threshold crossings (TCs) and spike band power (SPB) are quite correlated (this is also the case with previous studies we are aware of). We measured the correlation between SBP and TCs (binned every 10ms) on each electrode and found an average correlation across all electrodes of 0.59 ± 0.17 for T15 and 0.56 ± 0.21 for T16 (mean ± s.d.; p < 0.05), indicating substantial shared information.

To further examine the relationship between these features, we applied PCA to either TCs or SBP from [-750, 750] ms around speech onset. The resulting PCA projections are shown in **Response to Reviewers Figure 6** below. Qualitatively, both neural features yield similar clustering patterns, showing separable grouping by loudness levels and words. Quantitatively, the percentage of variance captured by the top three PCs differs: for T15, SBP captures [48, 10, 4]% and TC captures [21, 8, 5]%. A similar trend is observed for T16. This suggests that TCs contain fewer large-variance shared activity components, while SBP activity across the arrays is lower-dimensional (or at least has a large-variance first PC). This makes sense given that SBP is summing together spikes from a small vicinity around each electrode tip which amplifies the underlying neural factors that influence the population widely.

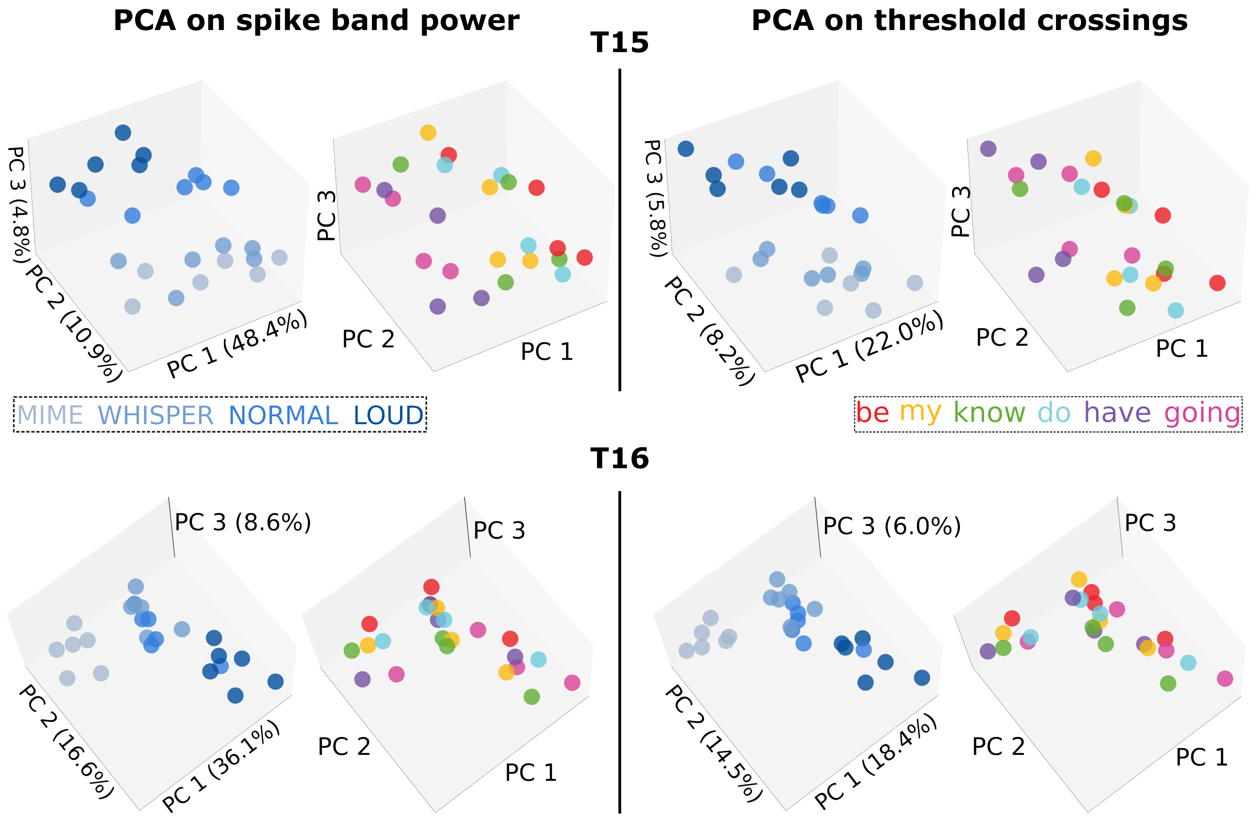

Response to Reviewers Figure 6: PCA data visualizations using spike band power and threshold crossings look qualitatively similar. The first 3 PCs of spike band power capture more task-related neural variance compared to threshold crossings.

We used SBP for the dimensionality analyses because in chronic Utah arrays, SBP tends to provide more information about population-level behavior (it detects more neurons overall because the smaller neurons that TCs miss tend to have information, so the overall SNR about the behavior of interest is higher) [Wilson 2020, Nason 2020]. We now make this choice of which signal to analyze explicitly clear in **Methods Section 4.3.4, line 494** as follows:

> To determine if the ensemble neural activity encoded attempted loudness, we performed PCA and demixed PCA[40] on the word-loudness task data (Fig. 2, Supp. Fig. 5). …We chose to perform these neural ensemble analyses on spike band power because it reflects the aggregate spiking activity of a number of neurons around each electrode and has been shown to capture more overall task-related neural activity in chronic human Utah array recordings than threshold crossings, likely due to the additional signal provided by detecting smaller neurons[24,41]. We note that the PCA and dPCA plots resulting from the following analyses when performed on threshold crossings looked very similar (not shown) and the threshold crossings and spike band power had correlations (mean ± s.d. across all electrodes) of 0.59 ± 0.17 for T15 and 0.56 ± 0.21 for T16.…

(In case you are wondering: we plot TCs firing rates in Figure 1 since spike rates are what most readers want to see for a more “raw” view of the neural activity, and note that we’ve now added

spike-sorted single unit results in the new Supplementary Figure 4 to provide even more of this.)

2. Relatedly, what do the ensemble coding dynamics look like if threshold crossings are used instead of spike band power?

We performed dPCA on threshold crossings from [-750, 750] ms around speech onset for T15 and T16 respectively (**Response to Reviewers Figure 7**). Similar to what we observed with spike band power (Fig. 2b-c, Supp. Fig. 5b-c), here we again observed that much of the variance in the neural activity is captured by the condition-independent component, reflective of the shared temporal modulations in the neural activity irrespective of the task condition. Neural projections also separated out according to different words and loudness levels. Overall, the dPCA results from threshold crossings followed the same trends observed with spike band power, with differences in just the specific demixed components that captured variance related to individual task parameters (which is expected). Given that these are large plots and the TCs results look similar to the SBP results already in the manuscript, we would prefer not to also add these largely-redundant TCs dPCA plots unless the reviewer or editor feels strongly they should be added.

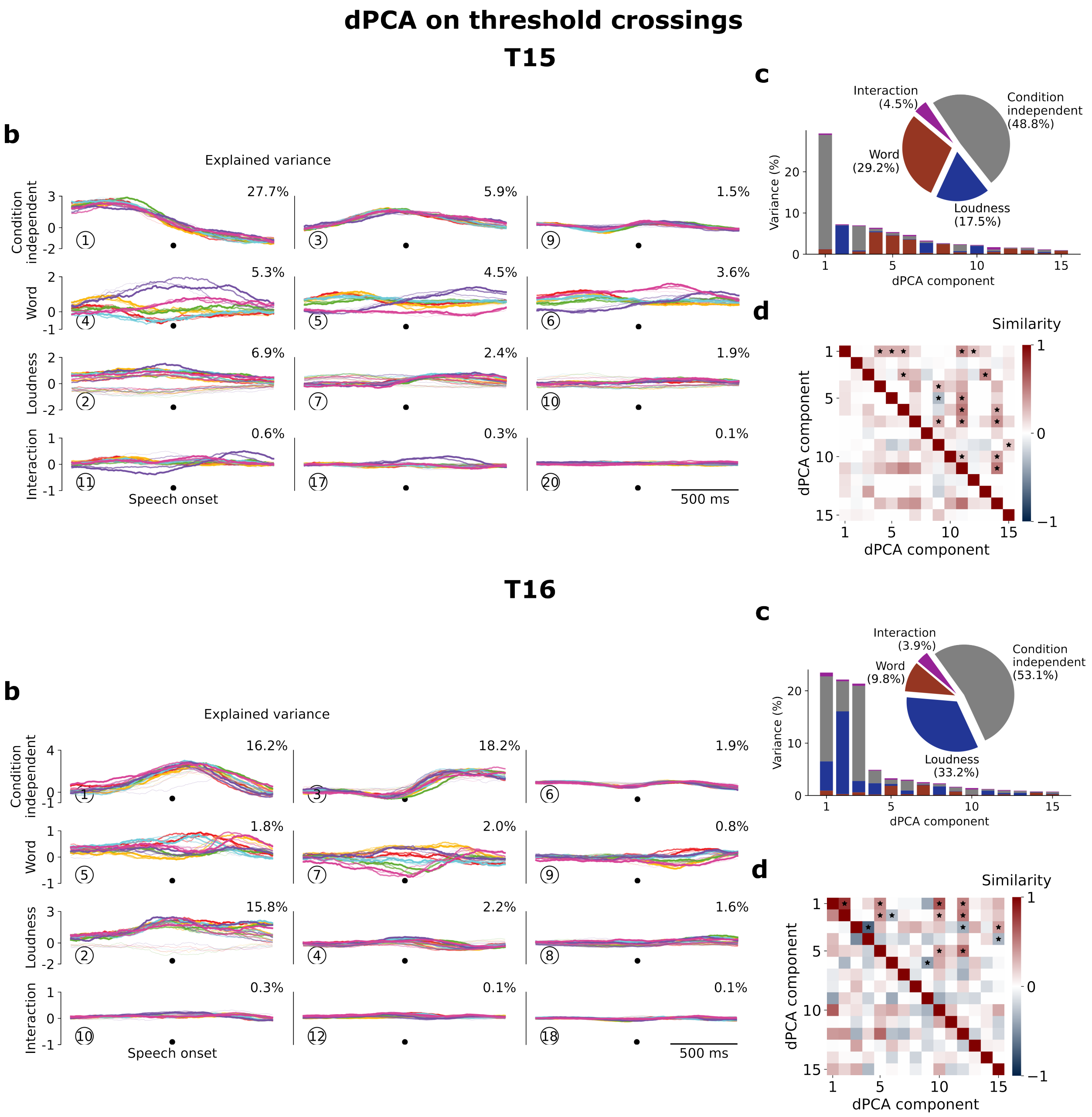

Response to Reviewers Figure 7: dPCA on threshold crossings results are similar to that using spike band power. Subpanels b, c, d, correspond to the same subpanels in Figure 2 and Supplementary Figure 5.

3. The authors emphasize that loudness could be decoded well before speech onset, indicating preparatory encoding. However, Figures 3 and Supplementary Figure 4 also show that decoding accuracy remains significant for up to ~2 seconds after speech offset in both participants. How should this sustained post-speech decoding be interpreted? Does it reflect residual motor activity, cognitive state signals (e.g., attention or effort), or a delayed return to baseline? A discussion of this phenomenon would be helpful.

We have addressed this phenomenon in **Shared Question #2** at the beginning of this document, where we present our hypothesis regarding why decoding persists after speech offset, along with an additional supplementary figure and corresponding revisions in the Discussion section.

4. In the BCI experiment, the participants performed two tasks simultaneously: modulating loudness while forming words. This may impose additional cognitive or motor demands, compared to the classical speech BCI paradigms. Did this dual-task paradigm affect the performance of the primary brain-to-text decoder, and if so, how was this evaluated?

In the closed-loop BCI experiment, we observed that the brain-to-text decoding had a mean word error rate of 3%. However, we now recognize that we should have contextualized these results (i.e., made clear that this is similar to our previously reported primary brain-to-text decoder accuracy). We have updated **Results section 2.4, line 254** as follows:

> The loudness-integrated speech BCI achieved a loudness classification accuracy of 94%, with a true positive rate of 89% and a false positive rate of 2% (**Fig. 4d**). We observed that despite the added cognitive and motor demands of modulating speech loudness in the sentence-loudness task, the 3% word error rate achieved was similar to previously reported brain-to-text accuracy for this participant[20]. However, it remains to be seen whether there are more subtle effects on word decoding if this were to be used when producing more complex sentences during day-to-day independent use.

5. The proportion of variance explained by word (22.7% for T15 vs. 8.1% for T16) and by loudness (13.9% for T15 vs. 32.2% for T16) differs substantially between participants, whereas other dPCA components appear comparable. Can the authors comment on potential reasons for this discrepancy?

To address this difference between the two participants, similar to the analysis presented in Fig. 1c and Supplementary Fig. 1b – where we identified electrodes tuned to loudness levels across vPCG – we conducted a comparable analysis to determine whether electrodes were tuned to different words. Specifically, we measured the number of significant differences in firing rates across pairs of attempted words using a one-way ANOVA with post-hoc Tukey's honestly significant difference test ($p < 0.05$).

For participant T15, electrodes with firing rates that significantly differed across word pairs were distributed throughout the vPCG region sampled by the arrays, closely resembling the spatial pattern observed for loudness encoding. Across T15's vPCG, 86% of electrodes showed significant modulation to both loudness levels and words (measured as the number of filled circles in the figure). In contrast, for participant T16, word tuning was primarily observed in the 6v array, whereas loudness tuning was evident in both the 6v and 55b/PEF arrays. As a result, only 63% of electrodes were tuned to words, while 85% were tuned to loudness. We summarize these results by each array in **Response to Reviewers Table 1 and 2**.

Consequently, we believe that the difference in the proportion of variance explained by word and loudness between participants arises from this underlying spatial distribution of tuning across vPCG. Specifically, in T15, both word- and loudness-related information were broadly represented across the sampled region, whereas in T16, loudness-related modulations were more widespread than word-related ones, leading to the observed discrepancy in the explained variance across these components.

T15's array	% of electrodes tuned to loudness levels	% of electrodes tuned to words
55b	82.8	92.2
d6v	75	84.4
M1	93.7	87.5
v6v	92.2	80
All	85.9	85.9

Response to Reviewers Table 1: Percentage of electrodes tuned to words and loudness in participant T15.

T16's array	% of electrodes tuned to loudness levels	% of electrodes tuned to words
55b/PEF	76.6	42.2
6v	93.7	82.8
All	85.1	62.5

Response to Reviewers Table 2: Percentage of electrodes tuned to words and loudness in participant T15.

We have made the following changes to the revised manuscript incorporating this new analysis. We added the word-tuning subfigures as **Fig. 1e** and **Supp. Fig. 1d** for participants T15 and T16, respectively.

e

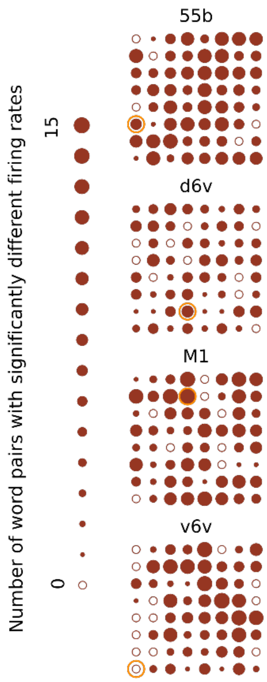

Figure 1. Loudness and word encoding across vPCG….e. Electrodes tuned to attempted words determined by significant differences in firing rates between words (one-way ANOVA with post-hoc Tukey's honestly significant difference test, $p < 0.05$)....

d

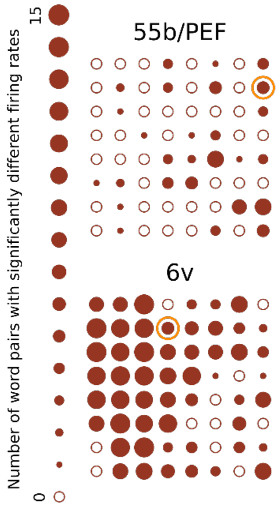

Supplementary Figure 1. Loudness and word encoding across participant T16's vPCG….d. Electrodes tuned to attempted words determined by significant differences in firing rates between words (one-way ANOVA with post-hoc Tukey's honestly significant difference test, $p < 0.05$)....

We have also updated the text in **Results section 2.1, line 104** describing these findings.

> …Electrodes with firing rates that significantly differed across pairs of attempted loudness levels ($p < 0.05$, one-way ANOVA with post-hoc Tukey's honestly significant difference test) were distributed throughout T15's vPCG region sampled by the arrays (**Fig. 1d**). Similar firing rate modulations were observed in T16: within her 55b/PEF array, electrodes that exhibited significantly different firing rates for different loudness levels were predominantly located on the putative 55b cortical region (**Supp. Fig. 1c**). Using the same statistical criterion, electrodes showing significant differences in firing rates across word pairs were also distributed throughout T15's vPCG region (**Fig. 1e**). Overall, 86% of electrodes showed significant modulation to both loudness levels and words. However, there was markedly less word tuning on T16's 55b/PEF array compared to the 6v array (**Supp. Fig. 1d**): 85% of electrodes were significantly tuned to loudness whereas only 63% were tuned to words.…

As for why there are these differences, with just n=2 participants we are not yet in a position to draw conclusions. We'd speculate that the specific location within 55b is different (in particular, T16's array is more anterior and closer to PEF).

6. PCA was performed using [-100, 400] ms around speech onset, while dPCA used [-750, 750] ms. What is the rationale for choosing different analysis windows, and could this influence the observed separability of word vs. loudness encoding?

We have addressed this point in **Shared Question #3** at the beginning of the document. Our choice of a shorter window for PCA was initially somewhat arbitrary, intended to focus on neural activity tightly centered around speech onset, whereas dPCA was performed over a wider window to capture broader temporal modulations. We have now shown that expanding the PCA analysis window to [−750, 750] ms yields similar results: the total variance explained by the top three PCs remains similar for both participants, and the relationships between different task conditions are preserved. For consistency, we have updated the manuscript to report the PCA results using the [−750, 750] ms window.

7. I understand that using threshold crossing facilitates online BCI decoding. But it would be informative to know whether spike sorting was attempted and, if so, how individual neurons encode speech loudness and mode.

We appreciate the reviewer's suggestion and agree that adding single unit activity results would add important basic neurophysiology results to the manuscript. We have now performed spike sorting to examine how individual neurons encoded the task parameters. We identified 237 and 122 neurons across vPCG for participants T15 and T16, respectively. For each neuron, we assessed significant differences in firing rates across loudness conditions and across word conditions in the same way as for threshold crossings (one-way ANOVA with post hoc Tukey's honestly significant difference test, $p < 0.05$), and classified neurons as tuned to word, loudness, both, or neither (see **Supplementary Figure 4**).

In participant T15, 65% of neurons were tuned to both words and loudness, 22.8% to words only, and 8% to loudness only. In participant T16, 50.8% of neurons were tuned to both, with a higher proportion tuned exclusively to loudness compared to words (27% vs. 6.6%). In both participants, only a small proportion of neurons were not tuned to either task parameter. This pattern aligns with the results from Fig. 2c and Supp. Fig. 5c, where the dPCA explained variance showed a similar trend for T15 and T16. These findings confirm that spike-sorted single units exhibit tuning consistent with the population-level trends observed in spike band power, and that the mixed tuning for both loudness and words was not a trivial consequence of threshold crossings or spike band power mixing together sparsely tuned individual neurons.

We have updated the manuscript with these details.

Supplementary Figure 4:

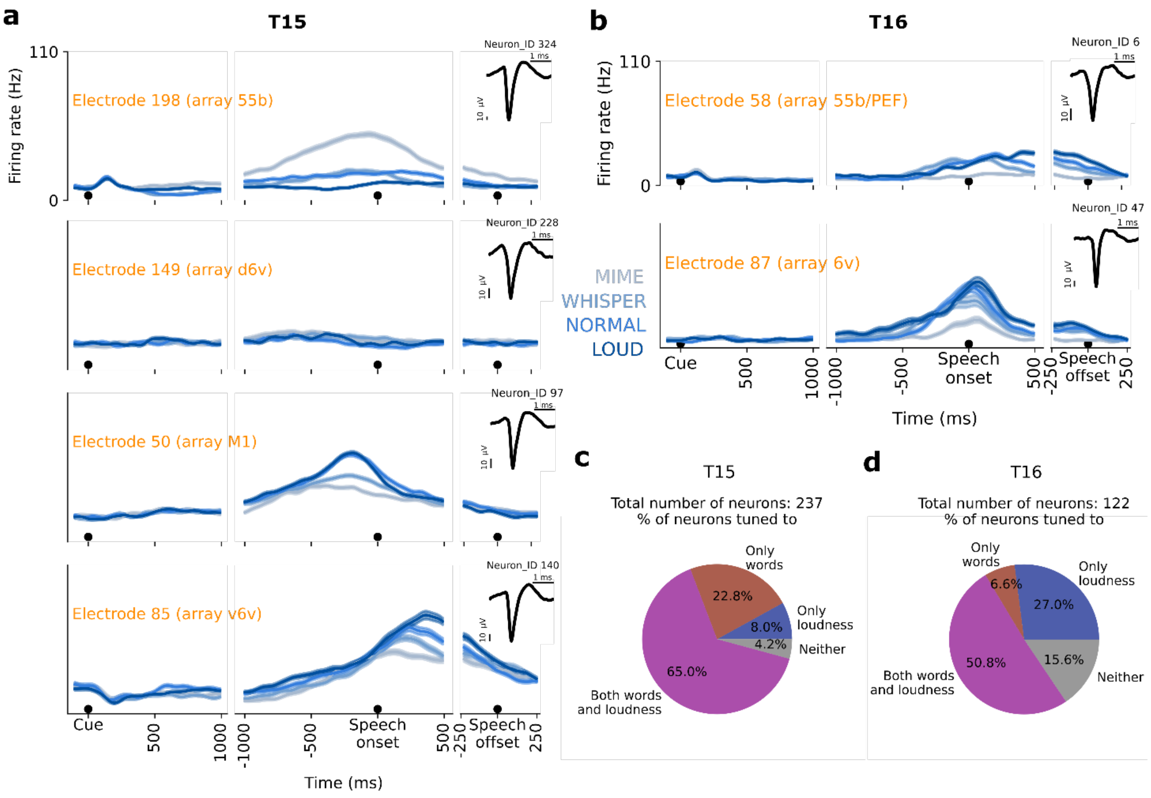

Supplementary Figure 4: Single-unit activity and task tuning. a,b) Mean firing rates (± s.e.) from an example neuron recorded from each array for participants T15 and T16, respectively. Insets show mean action potential waveforms of these neurons. **c,d)** Proportion of neurons tuned to different task parameters (word, loudness, both, or neither) for T15 and T16, respectively. Tuning was determined by measuring, for each neuron, significant differences in firing rates across loudness conditions and across word conditions (one-way ANOVA with post-hoc Tukey's honestly significant difference test, $p < 0.05$).

Methods section 4.3.8:

4.3.8. Spike sorting and single-unit analyses

To examine task tuning at the level of individual neurons (Supp. Fig. 4)., we performed spike sorting on the bandpass-filtered and denoised neural data using MountainSort5[43], implemented via the SpikeInterface framework[44] Candidate spike events were detected when the signal exceeded –4 times the median absolute deviation of the noise. Detected events were automatically clustered into putative single units using the default MountainSort5 pipeline, followed by manual curation to remove noise clusters and merge duplicate units.

To determine if a given neuron was tuned to behavioral conditions, for each neuron, we considered the firing rate from [-0.5, 0.5] s around speech onset. We then compared the firing rates between different pairs of loudness conditions or word conditions separately using the one-way ANOVA statistical test with post-hoc Tukey's Honestly Significant Differences test. Each neuron was then labeled as tuned to word, loudness, both, or neither, depending on the significance value across the two tests (Supp. Fig. 4c-d).

Results section 2.1, line 126:

Complementing these per-electrode threshold crossings analyses, we also examined task tuning at the level of individual neurons using spike-sorted single-unit activity (**Supp. Fig. 4**). For each neuron, we measured the firing rate from [-0.5, 0.5] s around speech onset and assessed tuning for word and loudness (one-way ANOVA, Tukey's honestly significant difference post- hoc test, $p < 0.05$). In T15's recordings, 65% of neurons were tuned to both words and loudness, 22.8% to words only, and 8% to loudness only (**Supp. Fig. 4c**). In T16's recordings, 50.8% of neurons were tuned to both, with a higher proportion tuned exclusively to loudness compared to words (27% vs. 6.6%) (**Supp. Fig. 4d**). Only a small fraction of neurons in both participants were not tuned to either behavior.

Minor:

1. In Figure 1d and Supplementary Figure 1c, a legend could be added to clarify how the size of the black circles corresponds to the number of loudness levels an electrode is tuned to.

We have added legends to **Figure 1d-e** and **Supplementary Figure 1c-d** to denote the depth of loudness or word tuning an electrode exhibits. **Figure 1d-e** are shown below as an example.

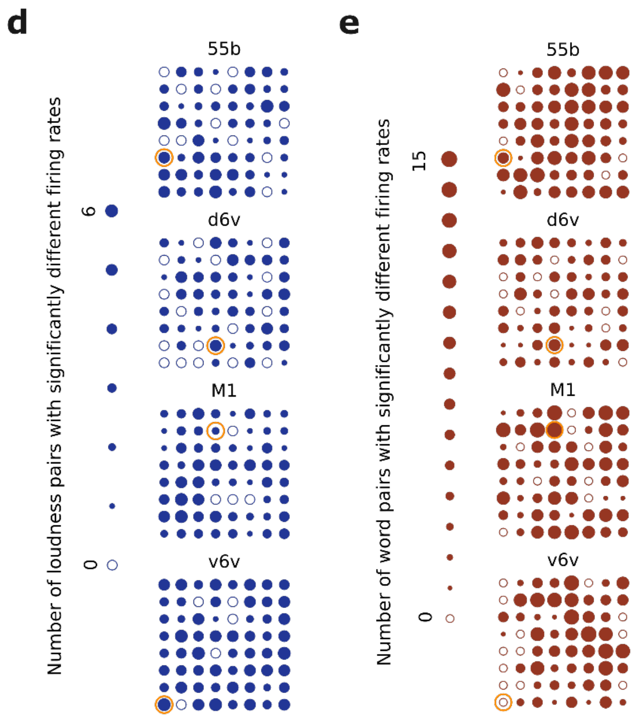

2. In Supplementary Figures 3a and 3b, it will be better to use consistent labels, e.g. breathe NORMALLY, breathe DEEPLY, NORMAL loudness level, LOUD loudness level.

Thank you for pointing out that the panel b horizontal label used “Instructed breath” rather than “breathe ____” and “Attempted loudness” rather than “____ loudness level” as in panel a, which is indeed inconsistent. We have now incorporated consistent labels as suggested in **Supplementary Figure 6a-b** (Please note that the supplementary figures have been reordered to match the order in which they are referenced in the updated Results section. Supplementary Figure 3 from the initial submission is now Supplementary Figure 6.):

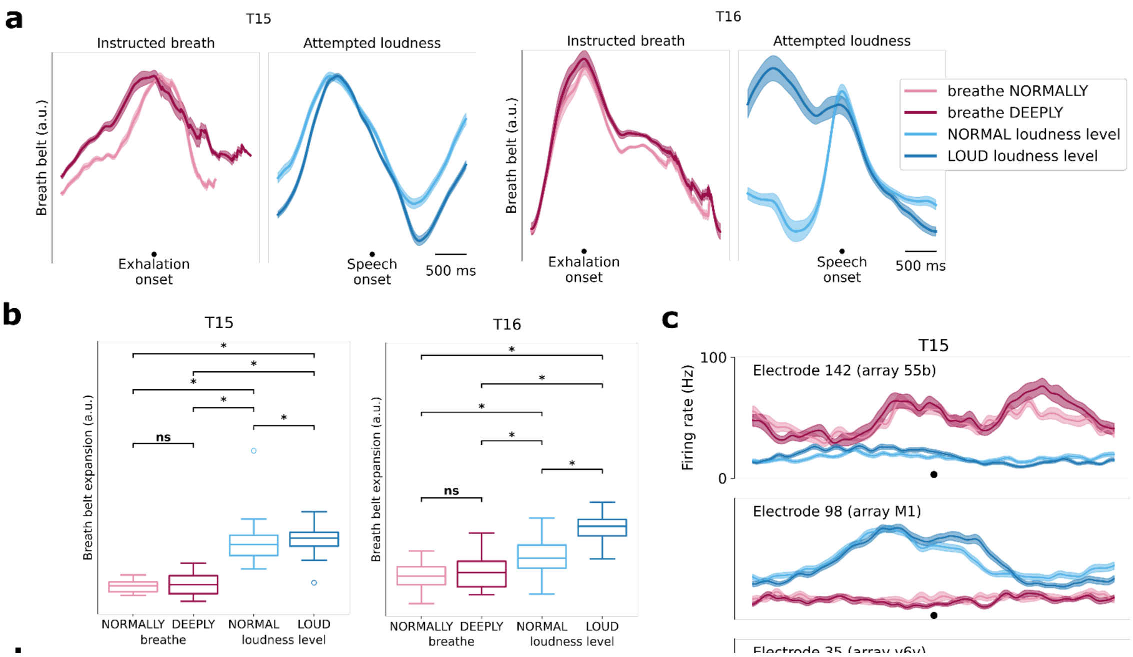

Reviewer #4 (Remarks to the Author):

In "Encoding of speech modes and loudness in ventral precentral gyrus", Srinivasan et al. use multiple intracortical electrodes along the precentral gyrus (PCG) to decode speech loudness levels from recorded neural population activity in two dysarthric participants. The work focuses on two main findings: 1) There is separability between the neural representations of loudness levels and words in ventral PCG (vPCG) and 2) loudness levels can be accurately decoded from neural activity in vPCG, specifically in closed-loop brain-computer interface (BCI) contexts. The authors achieve high decoding accuracies of loudness levels across both patients and show that loudness levels are robustly represented across many electrodes in their electrode arrays. The authors conclude that speech modes and loudness are well-represented in the neural activity of vPCG and that future speech BCIs can include this prosodic decoding to provide users with additional expression in their communication.

The authors achieve great success in decoding speech modes and loudness levels from vPCG. Specifically, they show impressive performance of a proof-of-concept speech loudness decoder that aims to extend speech BCIs beyond solely brain-to-text decoding that previous studies have shown. However, I feel that the current work does not sufficiently investigate the encoding of these speech modes and loudness levels in vPCG, as the title suggests. I think the core idea and result here is very interesting, but further work needs to be done to clarify the results/interpretation.

We thank the reviewer for their thorough assessment of the manuscript. We are glad they see significance in our findings regarding separable neural representations of words and loudness. We also appreciate the positive feedback on our proof-of-concept loudness decoding in a closed-loop speech BCI. We address individual questions below and believe that with these changes, as well as various other improvements in response to the other reviewers' feedback, the results and

interpretation are now more clear and there is more information presented about the encoding of speech modes and loudness.

1. While the authors have shown separability between word and loudness subspaces, the abstractness from constraining the analyses to only low-dimensional subspaces inhibits some understanding of how these representations differ. Are there unique spatial patterns associated loudness vs word representations (seems likely as the decomposition is performed on the electrode dimension)? Or certain neuroanatomical locations associated with these separable PCs? The authors could investigate this and provide further interpretability on the separability by associating electrodes in neuroanatomical space to the principal components (e.g. simply visualizing the weights of electrodes for a given PC/dPC associated with loudness or word information). An encoding model predicting spike band power from loudness or word features would provide similarly useful information.

To provide spatial interpretability for the encoding of words and speech loudness, we have now expanded **Figure 1** to show where on the electrode arrays there was tuning to words (red in the updated figure) and loudness (blue in the updated figure):

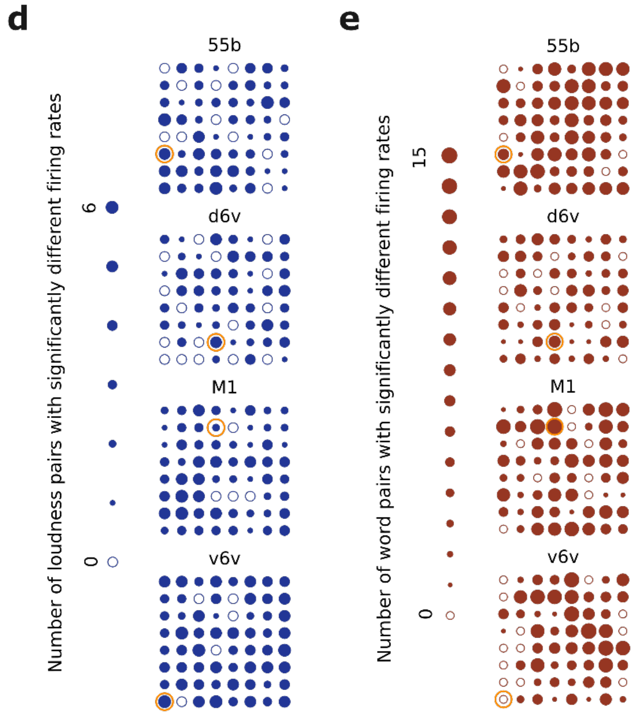

Figure 1. Loudness and word encoding across vPCG….d. Electrodes tuned to attempted speech loudness level, determined by significant differences in firing rates between loudness conditions (one-way ANOVA with post-hoc Tukey's honestly significant difference test, $p < 0.05$). **e.** Electrodes tuned to attempted words determined by significant differences in firing rates between words (one-way ANOVA with post-hoc Tukey's honestly significant difference test, $p < 0.05$)….

Supplementary Figure 1 shows this for T16:

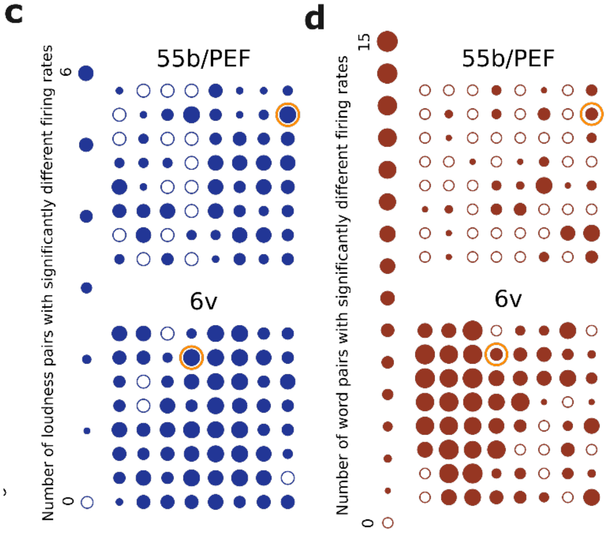

Supplementary Figure 1. Loudness and word encoding across participant T16's vPCG. …**c.** Electrodes tuned to attempted speech loudness level, determined by significant differences in firing rates between loudness conditions (one-way ANOVA with post-hoc Tukey's honestly significant difference test, $p < 0.05$). **d.** Electrodes tuned to attempted words determined by significant differences in firing rates between words (one-way ANOVA with post-hoc Tukey's honestly significant difference test, $p < 0.05$).

This reveals that both of these task parameters were encoded across the cortical areas examined (i.e., there's not a strict anatomical division). Our added sorted single unit analyses (summarized in **Supplementary Figure 4** and in **Results section 2.1**) further confirms that the division is not spatial at the scale of individual neurons on a given electrode. Thus, this separability of word and loudness encoding really is at the population activity (i.e., neural dimension) level.

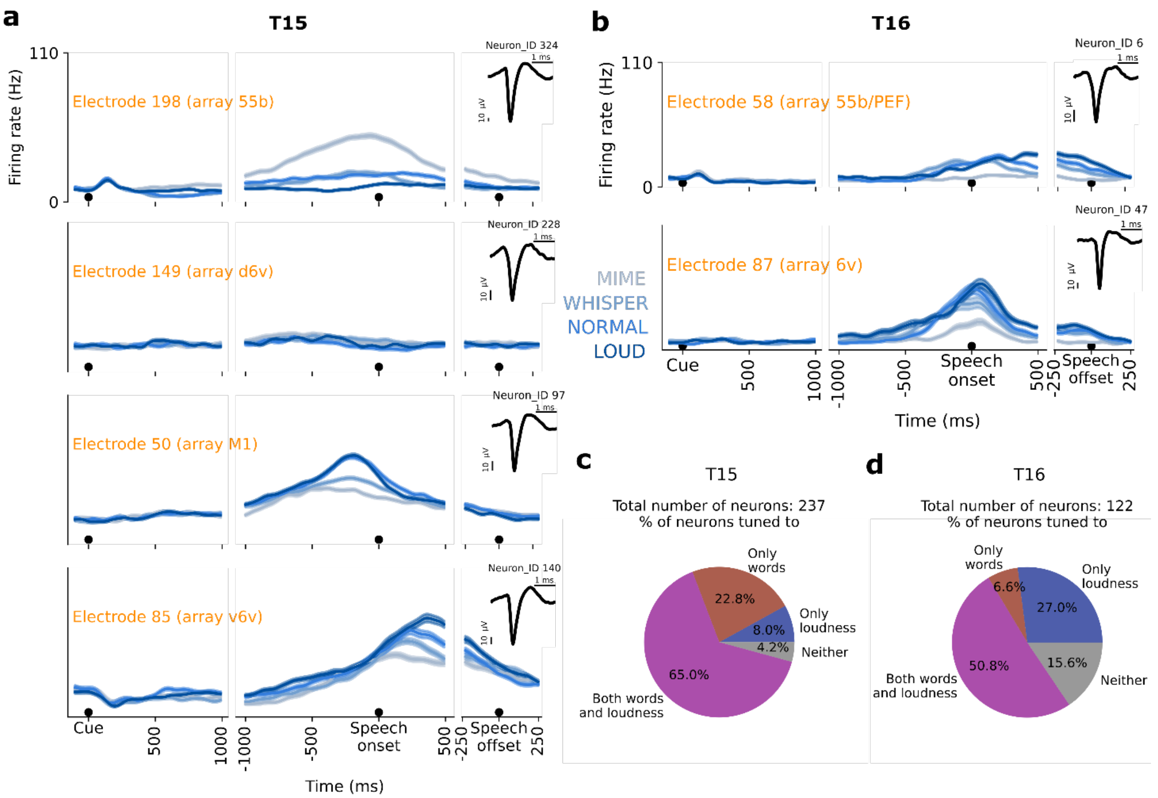

Supplementary Figure 4: Single-unit activity and task tuning. a,b) Mean firing rates (± s.e.) from an example neuron recorded from each array for participants T15 and T16, respectively. Insets show mean action potential waveforms of these neurons. **c,d)** Proportion of neurons tuned to different task parameters (word, loudness, both, or neither) for T15 and T16, respectively. Tuning was determined by measuring, for each neuron, significant differences in firing rates across loudness conditions and across word conditions (one-way ANOVA with post-hoc Tukey's honestly significant difference test, $p < 0.05$).

We believe these array map plots now showing tuning for both words and speech modes (computed for both threshold crossings in the main figures and also sorted single units in the supplemental figures) illustrate the spatial patterns observed across vPCG.

2. Relatedly, the somewhat orthogonal result is quite hard to interpret as presented. There are seemingly two distinct models for how the vPCG encodes loudness: one is that neurons (or populations) that are tuned to a specific word/feature fire work when the loudness increases, the other is that loudness is represented distinctly. It is unclear which of these (or both) are supported with the data given. A clearer result/interpretation would make the manuscript much stronger.

Thank you for pointing out that we need to be more clear in interpreting what the largely orthogonal neural dimensions mean for how loudness is encoded. We've updated the manuscript to do so in

two ways: (1) with new spike-sorted analyses to look at word- vs loudness-specific tuning at the single neuron scale, and (2) with updated text to more clearly interpret the results.

First, we have now included results from spike sorting. We found that in T15, 65% of neurons were tuned to both words and loudness, 22.8% to words only, and 8% to loudness only. In T16, 50.8% of neurons were tuned to both, with a higher proportion tuned exclusively to loudness compared to words (27% vs. 6.6%). Only a small fraction of neurons were untuned to either parameter. These results indicate that while some neurons jointly encode both task parameters, others are selectively modulated by either word identity or loudness, suggesting a mixed organization of feature selectivity in vPCG. These results have been added to the manuscript as **Supplementary Figure 4** and the last paragraph in **Results section 2.1**.

Supplementary Figure 4:

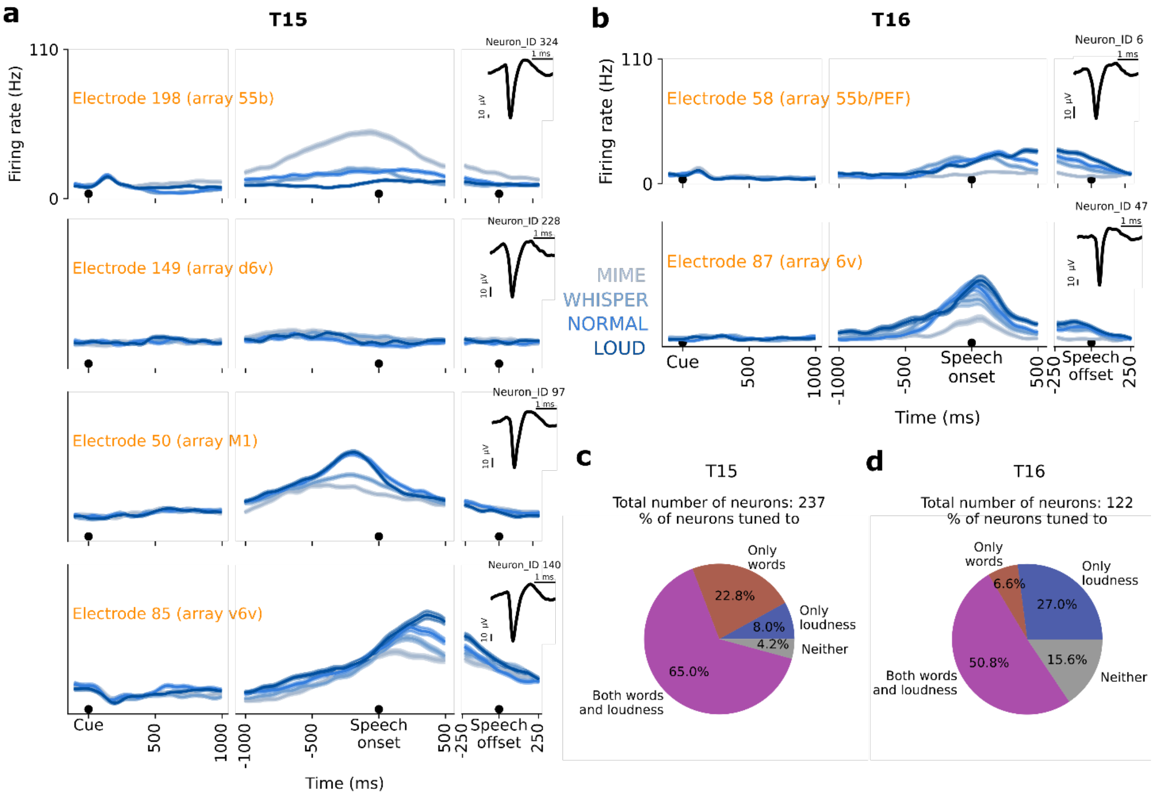

Supplementary Figure 4: Single-unit activity and task tuning. a,b) Mean firing rates (± s.e.) from an example neuron recorded from each array for participants T15 and T16, respectively. Insets show mean action potential waveforms of these neurons. **c,d)** Proportion of neurons tuned to different task parameters (word, loudness, both, or neither) for T15 and T16, respectively. Tuning was determined by measuring, for each neuron, significant differences in firing rates across loudness conditions and across word conditions (one-way ANOVA with post-hoc Tukey's honestly significant difference test, $p < 0.05$).

Results section 2.1, line 126:

> Complementing these per-electrode threshold crossings analyses, we also examined task tuning at the level of individual neurons using spike-sorted single-unit activity (**Supp. Fig. 4**). For each neuron, we measured the firing rate from [-0.5, 0.5] s around speech onset and assessed tuning for word and loudness (one-way ANOVA, Tukey's honestly significant difference post- hoc test, $p < 0.05$). In T15's recordings, 65% of neurons were tuned to both words and loudness, 22.8% to words only, and 8% to loudness only (**Supp. Fig. 4c**). In T16's recordings, 50.8% of neurons were tuned to both, with a higher proportion tuned exclusively to loudness compared to words (27% vs. 6.6%) (**Supp. Fig. 4d**). Only a small fraction of neurons in both participants were not tuned to either behavior.

Together with the orthogonality analyses reported below in response to the reviewer's next question, these results indicate that loudness and word representations in vPCG are largely distinct yet partly overlapping, consistent with a model where loudness is encoded along neural dimensions that are mostly separable from those representing word identity (i.e., much closer to the second of the two potential models the reviewer described).

We have added this interpretation to **Results section 2.2, line 177** to make it clearer:

> These findings suggest that word and loudness information in vPCG are represented in largely distinct, though partially overlapping, neural subspaces. This implies that a neuron could have strong tuning for different loudness conditions but not for different words, for words but not for loudness, for both (with different covariation patterns across neurons); or for neither.

3. The orthogonality analysis in Fig. 2d attempts to quantify separability between dPCs associated with word and loudness, however the conclusion is weakened by ambiguity in that the dPCs are "close to orthogonal". To strengthen this claim, the authors should consider additional metrics to quantify the lack of overlap between word and loudness representations specifically. Perhaps the authors could compare decoding performance when classifying loudness level or word identity from loudness and word dPCs?

We appreciate the reviewer highlighting this opportunity to add complementary analyses to strengthen the conclusions. We found the mean absolute pairwise cosine similarity between the top five word- and loudness-dPCs to be 0.14 for T15 and 0.28 for T16, indicating that these subspaces were not fully orthogonal (we have now added these quantifications to the text). As per the reviewer's suggestion, to further assess the degree of separation between word- and loudness-related neural representations, we performed a decoding analysis using projections onto specific subsets of dPCs (**Response to Reviewers Figure 8**). For T15, when decoding word identity using only the word-related dPC projections, classification accuracy was 98.5%, whereas loudness decoding from the same projections yielded 36.9% (only slightly above the 25% chance accuracy). Conversely, when decoding loudness level using only the loudness-related dPC

projections, accuracy was 95%, while word decoding from these projections was only 25.4% (again only slightly above the 16.6% chance accuracy). Using all dPCs together, word and loudness decoding accuracies were 97% and 92% respectively, very close to the accuracy values obtained using only the task-relevant dPC subset (e.g., loudness decoding from all dPCs vs. only loudness-related dPCs). Taken together, these results indicate that each task parameter is predominantly (and quite selectively) captured by its corresponding demixed components. A similar trend was observed for T16 (shown below). Given the large number of supplemental figures and that we can summarize these results efficiently in text, we have now described these results in the main Results text (copied for convenience later below) but not in figure form.

The modest above-chance decoding observed when using dPCs corresponding to the other task parameter reflects the fact that the dPC subspaces are not perfectly orthogonal, as shown in our orthogonality analysis in Figure 2d and Supp. Figure 5d. A small degree of shared variance between task parameters, such as co-modulation of neural activity by both word and loudness, results in weak but above-chance cross-decoding. These findings further support our characterization that word and loudness representations in vPCG are largely separable ("close to orthogonal") but not fully independent.

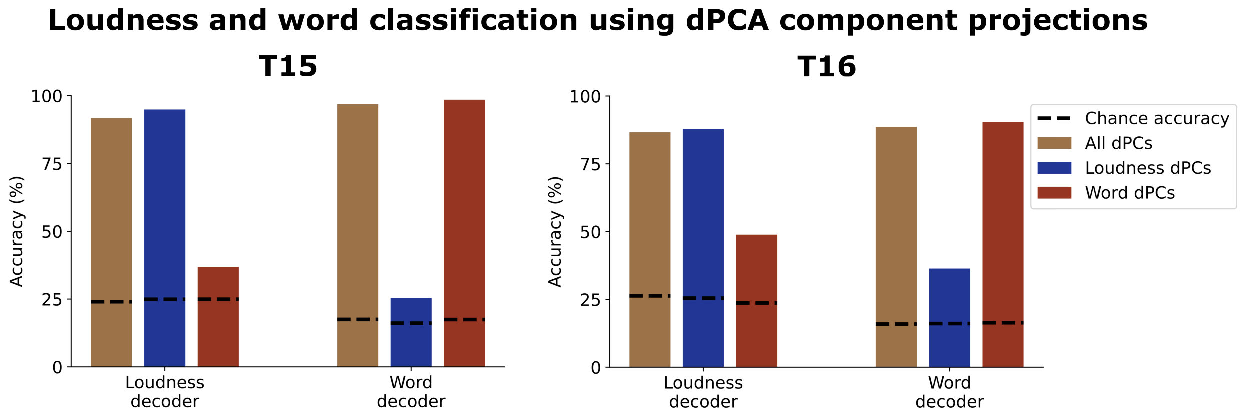

Response to Reviewers Figure 8: Loudness and word classification using different subsets of dPC projections.

We have made the following changes to **Results section 2.2, line 159**:

> Even though dPCA does not enforce orthogonality between encoding axes corresponding to different task parameters, most loudness and word dimensions turned out to be close to orthogonal (**Fig. 2d** and **Supp. Fig. 5d,** upper triangle; for example, dPCs 2 and 4, the largest T15 word and loudness dimensions, were close to orthogonal). The mean absolute pairwise cosine similarity between the top five word- and loudness-dPCs was 0.14 for T15 and 0.28 for T16. Control analyses found that the loudness-related neural differences could not be explained merely by differences in breath depth across conditions (**Supp. Fig. 6** and **Supp. Note 1**).

and the following changes to **Results section 2.3, line 212**:

Loudness encoding was robustly distributed across the neural ensemble. In a simulated neural drop-out analysis, we did not observe a substantial drop in decoding accuracy until decoding was attempted with fewer than ~30 electrodes (**Fig. 3d**, **Supp. Fig. 7d**). We also evaluated the performance of decoders trained on the word-specific and loudness-specific neural dimensions as a complementary method of quantifying the (low) degree of overlap in these neural subspaces. Using only the loudness-dPC projections (5-dimensional for T15, 7-dimensional for T16), loudness levels could be decoded robustly (95% accuracy for T15, 87.8% for T16; 25% chance). Similarly, words could be decoded with high accuracy using only the word-dPC dimensions (8-dimensional for T15, 5-dimensional for T16; 98.5% accuracy for T15, 90.4% for T16; 16.6% chance). In contrast, accuracies were only slightly above chance when we switched the projections used for decoding: 25.4% (T15) and 36.4% (T16) when decoding words from the loudness subspace, and 36.9% (T15) and 49% (T16) when decoding loudness from the words subspace.

4. Does the decoder have anything to do with the results from the first part of the paper? Or is it simply that the information is present and so it can be decoded online? The manuscript would benefit greatly if there was a stronger link between the first part of the paper (science) and the second (engineering).

We note that the connection between the scientific and engineering sections needs to be clearer. We view the primary focus of this work as establishing the basic science – that robust representations of speech modes and loudness exist in vPCG. The offline loudness decoding analyses (Results section 2.3) and subsequently the proof-of-concept online loudness decoding (Results section 2.4), were included to demonstrate that this information is not only present but can also be pragmatically decoded, in real-time, to start to add expressivity to speech BCIs.

We have revised the transition at the start of **Results section 2.3, line 182** to clarify this link by explicitly stating that the subsequent decoding sections builds on the scientific findings of loudness encoding and tests the feasibility of loudness decoding in closed-loop speech BCIs.

2.3. Offline decoding of loudness

We next evaluated whether loudness level could be accurately decoded from neural activity; our goal was to assess whether the loudness encoding in vPCG could be pragmatically used to increase the expressivity of a speech neuroprosthesis. To analyze how decoding performance evolved over time,...

5. Please include a label for the colorbar on Fig. 2d. While the upper and lower triangles show slightly different information, a label indicating the overall metric (e.g. “similarity”) would be appreciated.

Thank you for the suggestion. We have added “similarity” as the colorbar title in Fig. 2d and Supp. Fig. 5d.

6. Why are different time windows used for PCA vs dPCA decomposition? My interpretation was that PCA indicated different subspaces for word vs loudness information, and dPCA validated that by explicitly showing different components for word or loudness information. Using different time windows weakens the use of dPCA as a deeper investigation into the PCA representation.

We have addressed this difference in time windows in detail in **Shared Question #3** at the beginning of the document. In brief, we had used a shorter [-100, 400] ms window for PCA to obtain a concise view of neural activity around speech onset, a period in which loudness encoding is likely the strongest, before examining the full neural dynamics using dPCA. We showed that performing PCA on the extended [–750, 750] ms window around speech onset captures a similar amount of variance in the top three PCs. For consistency in the manuscript, we have updated Fig. 2a and Supp. Fig. 5a to show PCA results using the extended window.

7. In Fig. 3a, the authors approximately describe timepoints where loudness decoding increased above chance. The authors may consider the use of time-cluster permutation testing to identify temporal regions where loudness decoding significantly differed from chance to more accurately describe the timing of loudness information in vPCG.

Following the reviewer's suggestion, we have now determined the first time point at which loudness decoding performance was significantly above chance (time-cluster permutation test, $p < 0.05$). For T15, this occurred at 500 ms after cue onset, and for T16, at 330 ms after cue onset. These time points have been added to Fig. 3a and Supp. Fig. 7a, and the corresponding figure captions have been updated accordingly.

Figure 3a:

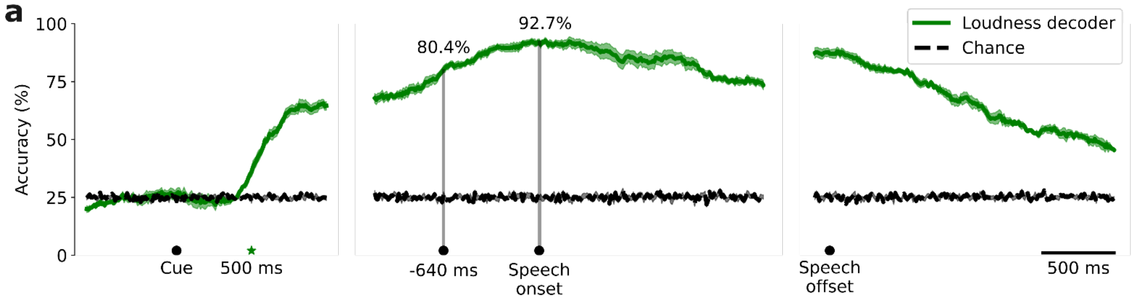

Figure 3: Loudness could be accurately decoded from neural activity offline. a. Loudness decoders were trained and evaluated on a 400 ms window of neural features with a 10 ms stride. Trial-averaged performance began to surpass chance at 500 ms after cue onset (green star) and decreased after speech offset (time-cluster permutation test, $p < 0.05$). Gray vertical lines mark when decoding accuracy exceeded 80% (640 ms before speech onset) and when maximum accuracy was achieved (at speech onset)....

Supplementary Figure 7a:

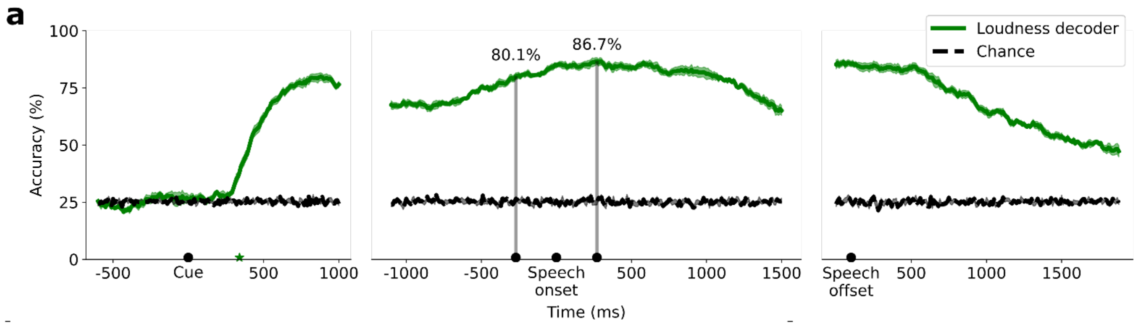

Supplementary Figure 7: T16's loudness could be accurately decoded from neural activity offline. a. Loudness decoders were trained and evaluated on a 400 ms window of neural features with a 10 ms stride. Trial-averaged performance began to surpass chance at 330 ms after cue onset (green star) and decreased after speech offset (time-cluster permutation test, $p < 0.05$).

We have also updated **Methods section 4.3.5, line 522**:

> For offline loudness decoding (Fig. 3, Supp. Fig. 7), we trained multinomial logistic regression models on smoothed neural features from the word-loudness task to predict the four loudness levels. A separate loudness decoder was trained and applied to 400 ms windows of neural features with a 10 ms stride (Fig. 3a, Supp. Fig. 7a). The decoding accuracy at every time point shown in the plots was obtained from a loudness decoder evaluated on the past 400 ms of neural features. The onset of above-chance decoding accuracy was identified using time-cluster permutation test….

8. Please state whether false discovery rate correction was used in cases with multiple significance tests (e.g. Fig. 4b).

Given Figure 4b is a graphical user interface, here we provide a response assuming the reviewer was referring to Figure 3b. The significance results reported in Figure 3b were obtained for each array through a simple permutation test. The mean decoding accuracy (averaged across folds) was compared against shuffled mean accuracies. p-value was computed as the number of times the decoding accuracy was lesser than shuffle accuracies. Since this analysis involved a single comparison per array (actual vs. shuffled mean accuracy), we did not apply a false discovery rate correction.

However, we have now used time-cluster permutation test to determine the temporal onset of significant decoding and updated Figures 3a and 7a and Methods section as mentioned above.

Reviewer #5 (Remarks to the Author):

I co-reviewed this manuscript with one of the reviewers who provided the listed reports. This is part of the Nature Communications initiative to facilitate training in peer review and to provide appropriate recognition for Early Career Researchers who co-review manuscripts.

Reviewer #5 (Remarks on code availability):

The github repository is well structured in separating code by different figure analyses and providing instructions on necessary packages for use. However, I was not able to run any of the analysis python files as described in the instructions. There are a few import errors that seem to be due to missing files ("from session_metadata import incorrect trials" or "from functions import get_audio_onset_offset", where session_metadata and functions appear to be non-package files that are not present). This prevented me from running downstream analysis files, as some relied on data saved from earlier files like psth.py. The code provided is useful for determining what analyses were performed and how exactly these analyses were implemented, but fixes should be made for users to run these analyses themselves on the sample data as the README describes.

We sincerely thank the reviewer for carefully evaluating the code repository and for their helpful feedback. We are glad to hear that the reviewer found that the repository is well organized and includes clear instructions for reproducing the analyses. We have updated the codebase and scripts such that one can now generate all the plots reported in the manuscript, as explained in **Shared Question #1**. All analyses will be fully replicable once the dataset is uploaded upon publication of the manuscript.

Response to Reviewers References

Wilson, G. H. et al. Decoding spoken English from intracortical electrode arrays in dorsal precentral gyrus. J. Neural Eng. 17, 066007 (2020).

Nason, S. R. et al. A low-power band of neuronal spiking activity dominated by local single units improves the performance of brain–machine interfaces. Nat. Biomed. Eng. 4, 973–983 (2020).
